# A hybrid nanopharmaceutical for specific-amplifying oxidative stress to initiate a cascade of catalytic therapy for pancreatic cancer

**DOI:** 10.1186/s12951-023-01932-0

**Published:** 2023-05-24

**Authors:** Fan Liu, Qinyanqiu Xiang, Yuanli Luo, Ying Luo, Wenpei Luo, Qirong Xie, Jingdong Fan, Haitao Ran, Zhigang Wang, Yang Sun

**Affiliations:** 1 Department of Ultrasound, The Second Affiliated Hospital of Chongqing Medical University, Chongqing, 400010 People’s Republic of China; 2 Chongqing Key Laboratory of Ultrasound Molecular Imaging & State Key Laboratory of Ultrasound in Medicine and Engineering, Chongqing Medical University, Chongqing, 400010 People’s Republic of China; 3 Department of Radiology, The First Affiliated Hospital of Chongqing Medical University, Chongqing, 400010 People’s Republic of China

**Keywords:** Nanopharmaceutical, Oxidative stress, Spontaneous cascade effect, STAT3, Contrast agent, Pancreatic cancer

## Abstract

**Background:**

Oxidative stress (OS) induced by an imbalance of oxidants and antioxidants is an important aspect in anticancer therapy, however, as an adaptive response, excessive glutathione (GSH) in the tumor microenvironment (TME) acts as an antioxidant against high reactive oxygen species (ROS) levels and prevents OS damage to maintain redox homoeostasis, suppressing the clinical efficacy of OS-induced anticancer therapies.

**Results:**

A naturally occurring ROS-activating drug, galangin (GAL), is introduced into a Fenton-like catalyst (SiO_2_@MnO_2_) to form a TME stimulus-responsive hybrid nanopharmaceutical (SiO_2_-GAL@MnO_2_, denoted SG@M) for enhancing oxidative stress. Once exposed to TME, as MnO_2_ responds and consumes GSH, the released Mn^2+^ converts endogenous hydrogen peroxide (H_2_O_2_) into hydroxyl radicals (·OH), which together with the subsequent release of GAL from SiO_2_ increases ROS. The “overwhelming” ROS cause OS-mediated mitochondrial malfunction with a decrease in mitochondrial membrane potential (MMP), which releases cytochrome c from mitochondria, activates the Caspase 9/Caspase 3 apoptotic cascade pathway. Downregulation of JAK2 and STAT3 phosphorylation levels blocks the JAK2/STAT3 cell proliferation pathway, whereas downregulation of Cyclin B1 protein levels arrest the cell cycle in the G2/M phase. During 18 days of in vivo treatment observation, tumor growth inhibition was found to be 62.7%, inhibiting the progression of pancreatic cancer. Additionally, the O_2_ and Mn^2+^ released during this cascade catalytic effect improve ultrasound imaging (USI) and magnetic resonance imaging (MRI), respectively.

**Conclusion:**

This hybrid nanopharmaceutical based on oxidative stress amplification provides a strategy for multifunctional integrated therapy of malignant tumors and image-visualized pharmaceutical delivery.

**Graphical Abstract:**

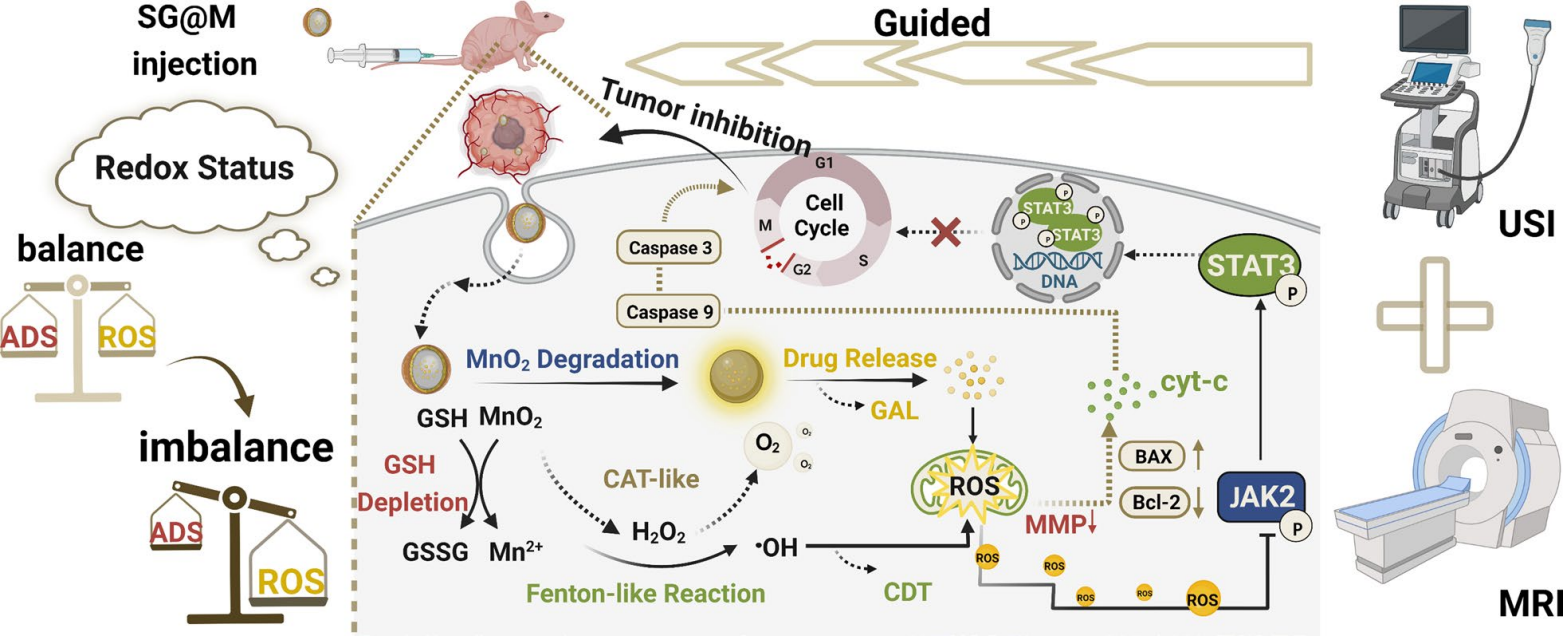

**Supplementary Information:**

The online version contains supplementary material available at 10.1186/s12951-023-01932-0.

## Introduction

Pancreatic cancer causes almost as many deaths as incidences due to its poor prognosis [1], with a 5-year survival rate of only 5% [2, 3]. It is anticipated that by 2025, pancreatic cancer will rank among the cancers that claim the most lives across several developed nations [1, 4]. New approaches to combat pancreatic cancer are needed since treatment remains challenging.

Reactive oxygen species (ROS), as oxidants, including singlet oxygen (^1^O_2_), superoxide radical (O_2_^‒^), hydroxyl radical (·OH) and hydrogen peroxide (H_2_O_2_), have made significant advances in physiology and disease as pleiotropic physiological signaling mediators [5]. The generation of ROS, one of the consequences of tumor oxidative metabolism [6], can be increased by a number of factors that promote tumor development, and this causes the ROS level in tumor cells to be greater than normal [7]. These increased ROS have different effects at different stages of tumor development; in brief, they both promote and suppress tumors, and this dual effect is closely related to the cellular antioxidant defense system (ADS). Although it is well known that excessive ROS can cause irreversible cell injury [8, 9], tumor cells respond by upregulating their ADS to ensure that ROS is limited to levels that do not induce cell death, that is, redox homeostasis [6, 9], thus allowing for proliferation [10]. At this point, ROS may be involved in tumor development as signaling molecule that activates the PI3K/AKT/mTOR and MAPK/ERK pathways [8, 11, 12]. Then, it is simple to comprehend that altering the redox balance to achieve a ROS-dominant position is possible, whether through an increase in ROS levels or a decrease in the cellular antioxidant capacity. This may activate oxidative stress (OS) [10] induced cell death and lead to the development of effective antitumor therapies [12, 13]. However, the two prevailing strategies for amplifying OS, ROS-inducing therapy and antioxidant-inhibiting therapy [8, 12], have not achieved an expected result individually. Development of chemotherapeutic drug resistance [8, 12] and ADS defense mechanisms [10, 14] have hampered the efficacy of OS. Therefore, combining the two strategies seems to be a highly promising approach to amplify OS.

A natural flavonoid, galangin (GAL) has exhibited anticancer behavior against various malignancies including breast cancer [15], ovarian cancer [16], gastric cancer [17], and hepatocellular carcinoma [18], but the potential mechanism for inhibition of pancreatic cancer has not been examined. Previous studies have shown that flavonoids are preferentially toxic to cancer cells, which are more sensitive to OS, while they are non-toxic to normal cells [19, 20]. This is because in cancer cells, flavonoids induce the redox cycle of with high concentrations of the major metal copper (Cu) in the nucleus [21], which is the most redox active metal ion in the biological system [22], leading to the formation of ROS that is overwhelming to ADS, resulting in apoptosis due to oxidative DNA breakage [19, 23]. However, the utilization of such flavonoids exhibiting pro-oxidant nature is limited by their low solubility, slow absorption rate and rapid metabolism in vivo [24–28]. Benefiting from the high therapeutic efficiency of multifunctional nanoplatform [29–31] and multiple nano-bio interactions, the combination of nanoagents and flavonoid holds promising potential for synergistic therapy of cancers [32, 33], either by improving its utilization or by reducing undesirable side effects.

Nanoagents targeting the most abundant ADS factor glutathione (GSH) [8] have demonstrated considerable effectiveness in malignant tumor therapy, among which manganese dioxide (MnO_2_) nanoagents have been widely explored due to their unique multifunctional properties. The intermediate valence state of Mn^4+^ in MnO_2_ gives the nanoagent a strong oxidizing capacity, allowing it to degrade quickly in a redox reaction with reducing compounds such as GSH, making it the ideal GSH-depleting agent [34–44]. The Mn^2+^ generated after degradation further mediates the Fenton-like reaction and catalyzes the conversion of excess H_2_O_2_ in the tumor microenvironment (TME) into toxic ·OH to achieve chemodynamic therapy (CDT) and enhanced T1-weighted magnetic resonance imaging (MRI) [45–49]. In addition, MnO_2_ also exhibits catalase-like (CAT-like) activity, which decomposes H_2_O_2_ to release oxygen (O_2_), thereby alleviating hypoxia [37, 38] as well as enhancing ultrasound imaging (USI) [50–52]. As a consequence, we envision a rational combination of GAL and such multifunctional nanoagents to construct a hybrid nanopharmaceutical that can multiply ROS while targeting ADS destruction to amplify OS impairment and operate as a multimodal imaging contrast agent to achieve dynamic visualization of nanopharmaceutical delivery processes.

**Scheme 1.**
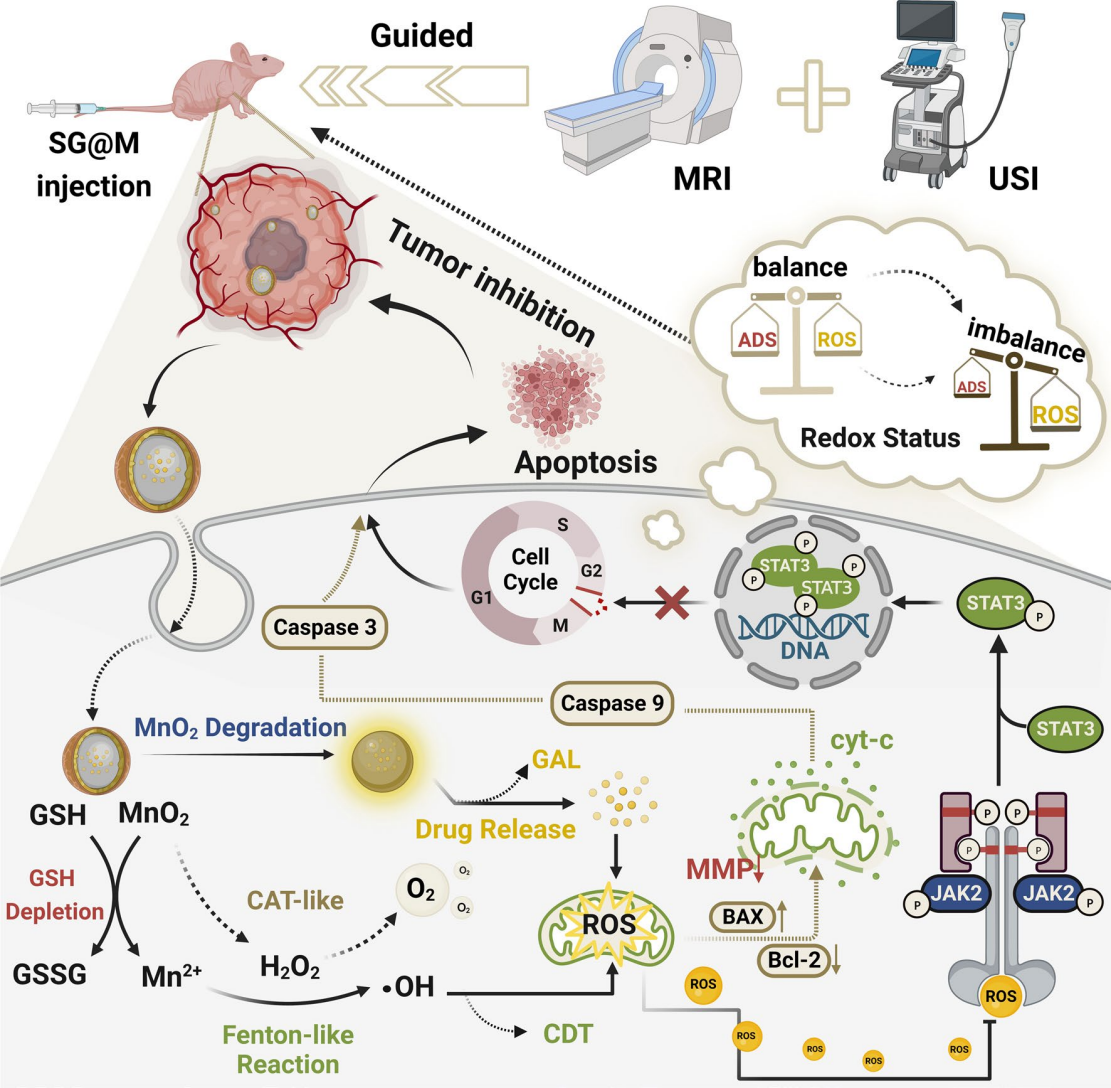
Schematic illustration of SG@M amplifying oxidative stress by breaking redox balance and inhibiting JAK2/STAT3 phosphorylation to suppress pancreatic cancer and enhance MR/US dual model imaging**.** (Created with www.BioRender.com)

For the above strategy, we introduced GAL into a self-decomposing silica core, which is known to be biosafe and widely used in drug loading systems [53, 54] to form SiO_2_-GAL nanospheres (denoted SG NPs). Then, the MnO_2_ layer was encapsulated in situ on the SiO_2_ surface to form the medically active SiO_2_-GAL@MnO_2_ nanospheres (denoted SG@M NPs) with the ability to disrupt redox homeostasis in cancer cells. As illustrated in Scheme 1, as a widely investigated nanosphere, the MnO_2_ shell consumes GSH and triggers a Fenton-like reaction, generating a massive amount of ·OH and Mn^2+^ to achieve CDT and T1-weighted MRI. When the MnO_2_ shell disappears, GAL is released from the degradable SiO_2_ carrier to stimulate ROS production. The excess ROS and weakened ADS shift the initial “redox balance” toward oxidative damage, inhibit the aberrantly activated JAK2/STAT3 pathway in pancreatic cancer [55–58] and induce apoptosis in PANC-1 cells. Equally promising, the CAT-like activity of MnO_2_ endows this nanopharmaceutical with the ability to be applied in USI and alleviate hypoxia in solid tumors due to the catalytic generation of O_2_ in the TME. In summary, based on the above principles, this hybrid nanopharmaceutical is expected to successfully inhibit the progression of pancreatic cancer.

## Methods

### Materials

3,5,7-trihydroxy-2-phenyl (Galangin, GAL) were purchased from Desite Biotechnology Co., Ltd. (Chengdu, China). Polyethylene Glycol (PEG, MW 600), ammonia hydroxide (28%) and dimethyl sulfoxide (DMSO) were obtained from TCI Development Co., Ltd. (Shanghai, China). Potassium permanganate (KMnO_4_), hydrogen peroxide (H_2_O_2_, 30%) and ethanol were provided by Chongqing Medical University. Tetraethyl orthosilicate (TEOS), methylene blue (MB) and doxorubicin (DOX) were provided by Aladdin Reagents Company (Shanghai, China). None of the compounds were further purified before usage.

### Preparation of SiO_2_-GAL nanospheres (SG NPs)

The SG NPs were prepared using a previously described procedure, with some alterations [54, 59]. Typically, ethanol (75 mL) and ammonia hydroxide (3 mL) were mixed and stirred for 0.5 h. Then, GAL (20 mg) was added into the liquid before stirring for another 0.5 h. To obtain SG NPs, TEOS (80 µL) as a source of SiO_2_ was added and stirred for 24 h, centrifuged at 13,000 rpm for 10 min, and washed three times with ethanol and water.

### Preparation of SiO_2_-GAL@MnO_2_ nanospheres (SG@M NPs)

According to the following equation: 2KMnO_4_ + H(OCH_2_CH_2_)_n_OH + O_2_ → MnO_2_↓ + HO(OCCOOH)n + K_2_MnO_4_ + H_2_O, we selected PEG as the reductant to react with KMnO_4_ to form the MnO_2_ shell [60]. Briefly, SG NPs (2 mg) were dissolved in deionized water (10 mL). After that, PEG (500 µg/mL, 200 µL) was added and mixed for 0.5 h to achieve complete fusion. The same volume of aqueous KMnO_4_ (200 µg/mL, 10 mL) was mixed and stirred for 24 h before centrifugation at 13,000 rpm for 10 min.

### Characterization

The morphology of the SG NPs and SG@M NPs was obtained using a Talos F200S TEM (Thermo Fisher Scientific CDLtd, CZ) and the Si and Mn elemental distributions were obtained by energy dispersive X-ray spectroscopy (EDS) using 4 in-column Super-X Detectors. A particle sizer and zeta potential analyzer (NanoBrook Omni, Brookhaven Instruments, UK) was applied to determine the particle size and zeta potential. X-ray diffraction (XRD) analysis was carried out using a D8 Advance X-ray diffractometer (Bruker, Germany). X-ray photoelectron spectroscopy (XPS) measurements of the valence states of Si and Mn were obtained using the K-Alpha XPS System (Thermo Fisher Scientific, Waltham, MA, USA). Fourier transform infrared (FT-IR) spectra of functional group information was captured with a Thermo Scientific Nicolet IS10. All of the samples were freeze-dried to powder form prior to analysis. UV–Vis spectra of MnO_2_ present in SG@M was detected using a Shimadzu spectrophotometer UV-3600. Each sample was digested using the hydrofluoric acid solution, and then the content of Mn and Si in SG@M were measured using inductively coupled plasma mass spectrometry (ICP-MS, PerkinElmer NexION 300X). Loading content of GAL was detected by high performance liquid chromatography (HPLC, Shimadzu LC-20AD) under the following chromatographic conditions: chromatographic column: Ultimate XB-C18 (4.6 × 100 mm 3 µm), mobile phase: 0.1% phosphate solution-methanol (30:70), flow rate: 1.0 mL/min, column temperature: 30 ℃, wavelength: 267 nm.

### MnO_2_ degradation and GSH depletion

SG@M (100 µg/mL, 1 mL) was dispersed in phosphate buffer saline (PBS, pH = 6.5), which included various concentrations of GSH (0, 1, 2, 3, 4, 5 mM). Then, the UV−Vis absorption spectra of these examples were measured, while simultaneously taking digital photos. The absorption spectra of SG@M dispersed in PBS (pH = 7.2) with or without GSH were additionally examined.

Extracellular GSH depletion was discovered using the 5,5′-Dithiobis-(2-nitrobenzoic acid) (DTNB) solution. In summary, SG NPs (100 µg) and SG@M NPs (100 µg) were dissolved in PBS (1 mL) and reacted with GSH (5 mM), respectively. Afterward, the sediment was removed by centrifugation (13,000 rpm, 10 min) for various incubation durations (0, 10, 30 min, 2, 12, 24 h). After adding the supernatant and DTNB solution (3 mg/mL, 10 µL) to a 96-well plate, the absorbance at 412 nm was measured.

### ·OH generation detection

Methylene blue (MB) identified the production of ·OH by SG@M. First, different concentrations of SG@M (0, 40, 80, 120, 240 µg/mL) were added to a buffer solution containing NaHCO_3_ (25 mM) and GSH (5 mM) to react for 15 min, and the supernatant was obtained by centrifugation. Second, the supernatant, as the reaction product was mixed with MB (10 µg/mL) and H_2_O_2_ (8 mM) and reacted for 30 min at 37 ℃. Finally, the absorbance of MB was measured at 665 nm. Alternately, the absorbance change of MB at 665 nm was also measured after an equivalent amount of SG@M (120 µg/mL) was reacted with different concentrations of GSH (0, 0.5, 1, 2, 5 mM).

### Mn^2+^ and GAL release

To imitate various biological conditions, SG@M (5 mg/mL, 1 mL) was placed into a dialysis bag, which was subsequently placed in 49 mL of PBS (pH 7.2, pH 6.5 with or without GSH (5 mM)). The Mn^2+^ and GAL were released with continuous shaking at 37 ℃. After that, the solution (6 mL) was taken outside the dialysis bag to measure the Mn^2+^ concentration by ICP-MS (5 mL) and the drug concentration by HPLC (1 mL) at various times (0, 2, 4, 6, 8, 10, 12, 24 h). After each sampling, the simulated solution (6 mL) was added to keep the volume constant. The cumulative release rate of Mn^2+^ in 2 h (1, 2, 4, 8, 16, 32, 60, 90, and 120 min) was measured according to the same method.

### O_2_ generation detection

To track O_2_ production, [Ru(dpp)_3_]Cl_2_ (RDPP) functions as a sensitive fluorescence probe that can be quenched by O_2_ [61]. First, each substance, SG (200 µg/mL, 1 mL) and SG@M (200 µg/mL, 1 mL) suspended in PBS (pH = 6.5) was treated with RDPP (50 µL) and H_2_O_2_ (40 µM, 200 µL). Then, the fluorescence intensity was recorded at 615 nm at various times (0, 2, 4, 6, 8, 10, 12 min). As a control, the O_2_ generation of SG@M (200 µg/mL) and free H_2_O_2_ were also measured. Every 6 s, a dissolved oxygen meter (JPBJ-608, Shanghai Oustor Industrial Co.) measured the dissolved oxygen content in the above groups.

### Cell culture

Human pancreatic cancer cell (PANC-1) was generously offered by the Stem Cell Bank, Chinese Academy of Sciences, and cultured in Dulbecco's modified eagle medium (DMEM) medium containing 10% fatal bovine serum (FBS) and 1% penicillin–streptomycin at 5% CO_2_ and 37 °C.

### Intracellular ROS generation detection

PANC-1 cells were exposed to free GAL, SiO_2_, SG, S@M and SG@M for 24 h. After 24 h, each group was introduced to 2,7-dichlorodihydrofluorescein diacetate (DCFH-DA) (10 µM) for 20 min of incubation before images were captured by laser scanning confocal microscopy (LSCM). Control cells were those that received no treatment. With ImageJ software, the fluorescence intensity was quantitatively analyzed.

### Mitochondrial membrane potential (MMP) detection

The changes in MMP in cells were assayed by JC-1 following various treatments. Briefly, PANC-1 cells were seeded in confocal dishes for 24 h, then incubated for another 24 h with either PBS, free GAL, SiO_2_, SG, S@M or SG@M. All cells were washed with PBS before being stained for 20 min with JC-1 solution (10 µM). LSCM obtained fluorescent images, and flow cytometry measured the ratio of JC-1 aggregates/monomers.

### In vitro therapeutic efficacy

For the CCK-8 assay, after PANC-1 cells (1 × 10^4^ cells per well) were seeded into a 96-well plate and incubated for 24 h, the medium was replaced with new medium containing SiO_2_, SG, S@M and SG@M at varying concentrations (0, 100, 200, 400 µg/mL), and further incubated for 24 h. Then, the cell viability was measured by CCK-8 assay. Cells were treated with different concentrations of S@M (0, 6.25, 12.5, 25, 50, 100, 200, 400 µg/mL) for 24 h and 48 h to further investigate the cytotoxicity of CDT. The cell viability was calculated to follow the Eq. (1) [38], and the additive therapeutic efficiencies of independent treatments were estimated using Eq. (2) [62, 63]:

1 $$Cell\,viability = \frac{{Absorption\,of\,post\,treatment}}{{Mean\,absorption\,of\,control}}\,\,\,\times 100\%$$

2 $$T_{additive} = \,100 - \left( {f_{SG} \times f_{CDT} } \right) \times 100,$$

where *f* is the fraction of surviving cells after each treatment.

To assess apoptosis, cells (2 × 10^5^ cells per well) were cultured in a 6-well plate for 24 h. Following several treatment methods employing PBS, SiO_2_, free GAL (22.18 µg/mL), SG (200 µg/mL), S@M (200 µg/mL) and SG@M (200 µg/mL), cells were collected for flow cytometry.

To assess relative Caspase 9 and Caspase 3 activity**,** a total of 3 × 10^6^ cells per group were coincubated for 24 h with each of the following 6 groups: PBS, SiO_2_, GAL, SG, S@M, and SG@M. Cell precipitates were obtained through centrifugation and digestion. According to the operating manual of the Caspase 9 Activity Kit and Caspase 3 Activity Kit (Beyotime), Caspase 9 and Caspase 3 activity were calculated for each group.

To assess cell cycle, PBS, free GAL (22.18 µg/mL), SG (200 µg/mL), S@M (200 µg/mL) and SG@M (200 µg/mL) were added to PANC-1 cells (2 × 10^5^ cells per well), which were then incubated for 24 h. For the flow cytometry experiment, cells were collected and dispersed in a solution of PBS (100 µL) and 75% ethanol (900 µL).

Western Blot analysis**.** PANC-1 cells were digested and centrifuged after 24 h of different treatments, washed 2–3 times with PBS and lysed in RIPA lysis buffer (cat. No. BL504A, Biosharp) with protease inhibitors to extract total proteins. The protein samples were then separated by SDS–PAGE and transferred to PVDF membranes (cat. No. IPVH00010, Millipor). Afterward, the membranes were blocked with 5% BSA solution for 1 h and incubated with primary antibodies against STAT3 (1:1000 v/v, GB11176, Servicebio), p-STAT3 (1:1000 v/v, AF3293, Affinity), p-JAK2 (1:1000 v/v, YP0155, Immunoway), Bcl-2 (1:1000 v/v, BF9103, Affinity), BAX (1:1000 v/v, GB11690, Servicebio), Cyclin B1 (1:1000 v/v, AF6168, Affinity), and GAPDH (1:5000 v/v, AF7021, Affinity) overnight at 4 ℃. After washing with TBST, the membranes were incubated with respective secondary antibodies conjugated with horseradish peroxidase for another 1 h at RT. Finally, the protein signals were recorded and visualized using hypersensitive ECL chemiluminescence reagent (Biosharp).

### Animals and tumor models

BALB/c nude mice (male, 5−6 weeks of age, 16−18 g body weight) were obtained from Tengxin Biotechnology Co., Ltd. (Chongqing, China) and housed in the Laboratory Animal Center of Chongqing Medical University. All animal experiments and procedures were approved by Ethics Committee of the Second Affiliated Hospital of Chongqing Medical University. By co-inoculating of PANC-1 cells with the Matrigel mixture, the pancreatic xenograft subcutaneous transplantation tumor model was established. Briefly, PANC-1 cells (1 × 10^7^ cells) were suspended in a PBS (100 µL) and Matrigel mixture (1:1, v/v) and subcutaneously transplanted into the right foreleg axilla of each mouse. After approximately 15 days, the tumor volumes of the tumor-bearing mice reached 100 mm^3^ for in vivo treatment and imaging. (The tumor volumes were calculated using the formula: 0.5 × length × width^2^).

### US imaging in vitro and in vivo

For in vitro US imaging, various solutions (H_2_O_2_, SG + H_2_O_2_, SG@M, SG@M + H_2_O_2_) were added to a transparent gel tube and then monitored via a Vevo LAZR imaging system (Visual Sonics Inc., Toronto, ON, Canada). For in vivo US imaging, SG@M NPs (10 mg/mL, 100 µL) were intravenously administered into PANC-1 tumor-bearing nude mice (n = 3). Prior to the injection as well as at certain intervals (30 min, 2, 4, 5, 6, and 24 h), B-mode and CEUS images were captured using the US system of EPIQ 7 (Philips, Netherlands).

### MR imaging in vitro and in vivo

Both in vitro and in vivo MRI were performed by the Siemens 3 T MRI system at The Second Affiliated Hospital of Chongqing Medical University. In vitro, different concentrations of SG@M (0.0625, 0.125, 0.25, 0.5, 1, 2 mg/mL) were distributed in a PBS solution (pH = 6.5) containing GSH (5 mM), and the samples were subsequently imaged by the MRI system. In addition, the same concentrations of SG@M NPs in PBS solution (pH = 7.2, without GSH) were utilized as a control. In vivo, SG@M NPs (10 mg/mL, 100 µL) were intravenously injected into PANC-1 tumor-bearing nude mice (n = 3), and T1-weighted signals were recorded before and after the injection for 2, 4, 6, 8, 12, and 24 h using the MRI scanner.

### Mn accumulation in tumor tissues and blood circulation

A dose of SG@M (10 mg/mL, 100 µL) was injected intravenously into PANC-1 tumor-bearing mice (n = 3). Approximately 20 µL of blood was taken from each tail at various times (2, 4, 6, 8, 12, 24 h) following injection. The mice were killed at the conclusion of the experiment, and the tumors were collected and weighed. ICP-MS was utilized to determine the quantity of Mn in each sample.

### Antitumor therapy in vivo

The mice were randomly assigned to 6 groups (n = 6) when tumor sizes reached 100 mm^3^: control (group 1); SiO_2_ (group 2); GAL (group 3); SG (group 4); S@M (group 5); SG@M (group 6). In the experimental groups, mice were intravenously injected with GAL (1 mg/mL, 100 µL), equal amounts of SG, S@M and SG@M (10 mg/mL, 100 µL), and the control group only received saline solution. On a regular basis throughout the course of the treatment, the body weight and tumor volume of the mice in each group were noted. On the 18th day, the mice were sacrificed, and the tumor tissues were dissected and stained with hematoxylin and eosin (H&E), terminal deoxynucleotidyl transferase dUTP nick end (TUNEL), and Ki-67 antibody. Meanwhile, immunofluorescence staining was performed on the harvested tumor sections to assess the expression using HIF-1α, JAK2, and p-STAT3.

To test the toxicity of SG@M NPs in vivo (n = 3), mice were intravenously injected with SG@M NPs (10 mg/mL, 100 µL). For H&E staining and hematological analysis, the primary organs and blood samples were collected at 0, 2, 4, 10, and 18 days after injection.

### Statistical analysis

All data are expressed as the mean ± standard deviation. One-way ANOVA tests and Student’s *t*-test were used to test the significance of differences among groups. Additionally, the statistical significance difference was shown as *p < 0.05, **p < 0.01, and ***p < 0.001.

## Results and discussion

### Synthesis and characterization of hybrid nanopharmaceutical

**Fig. 1:**
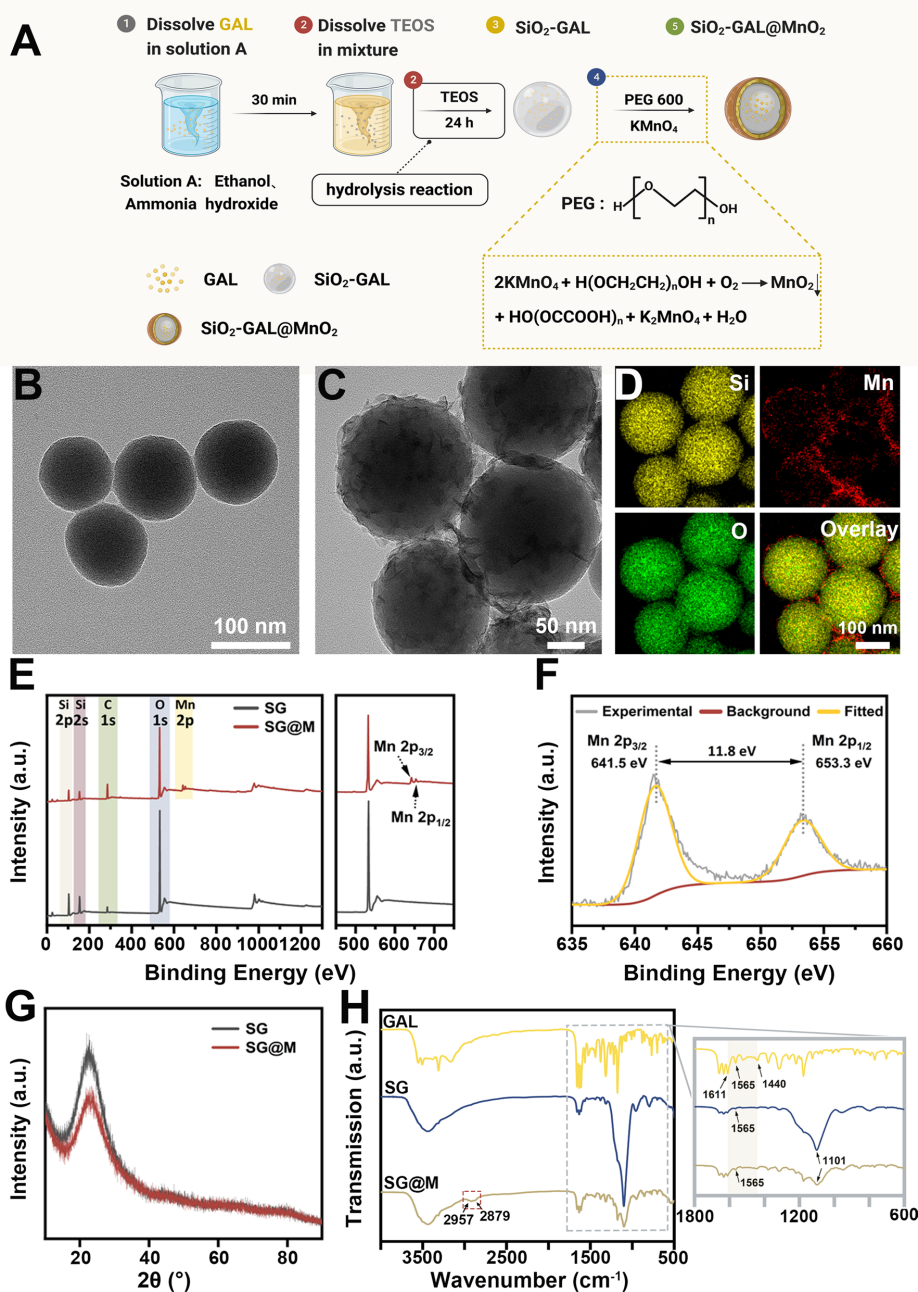
Preparation and characterization of SG@M NPs (Created with BioRender.com). **A** Schematic illustration of the synthesis of SG@M. **B**, **C** TEM images of SG and SG@M NPs and **D** the corresponding elemental mapping images. **E** XPS survey spectra and XPS spectra of **F** Mn 2p. **G** XRD spectra of SG and SG@M. **H** FT-IR spectra of GAL, SG, SG@M and local enlargements

As illustrated in Fig. 1A, SG NPs were formed using a conventional method [54, 64] by condensation of tetraethyl orthosilicate (TEOS) grown on the “seed” GAL surface under alkaline conditions. To prevent earlier drug leakage and respond to TME, a MnO_2_ layer was formed in situ on the surface of SiO_2_ via a reduction reaction of PEG and KMnO_4_. Transmission electron microscopy (TEM) images showed the spherical structure of SG NPs (Fig. 1B). After MnO_2_ coating, an irregular film structure appeared on the smooth spherical surface of SG NPs (Fig. 1C), with an increase in particle size from 124.45 ± 5.01 nm to 188.60 ± 6.49 nm and a change in zeta potential from − 20.77 ± 1.66 mV to − 26.60 ± 1.25 mV (Additional file 1: Fig. S1). The coated MnO_2_ shell was successfully encapsulated on the facade of SG NPs, as shown by the elemental mapping pictures of Si, Mn, and O (Fig. 1D). The XPS results revealed two typical binding energy peaks for MnO_2_ at 653.3 eV (Mn 2p_1/2_) and 641.5 eV (Mn 2p_2/3_), as illustrated in Fig. 1E and F. There were two diverse peaks for O 1 s (529.2 and 532.2 eV) that may result from MnO_2_ and SiO_2_ (Additional file 1: Fig. S2), and C 1 s and Si 2p spectral results proved the element valence information of SG@M NPs in Additional file 1: Fig. S2. Moreover, although no distinct peaks were seen in the XRD patterns following the coating of SG NPs with MnO_2_ (Fig. 1G), a notable decrease in peak intensities was observed, suggesting that the MnO_2_ layer was amorphous [34]. Additionally, a maximum absorption of MnO_2_ at 370 nm was observed in the UV–vis absorption spectra of SG@M (Additional file 1: Fig. S3). Further analysis using FT-IR spectroscopy showed that GAL was loaded during the synthesis of SG@M. The peaks in the region of 1440 cm^‒1^ to 1611 cm^‒1^ in the FT-IR spectra (Fig. 1H) were attributed to the conjugated benzene ring-type structure of GAL (Additional file 1: Fig. S4), and the peak at 1101 cm^‒1^ was attributed to Si−O−Si. Since PEG was used as the reduction, the MnO_2_-coated NPs showed a series of peaks of C−H bonds (2879 cm^‒1^, 2957 cm^‒1^) [59]. The above characterizations showed that SG@M was successfully synthesized, and the loading of GAL was estimated to be 11.09% based on the standard curve and measurement by HPLC (Additional file 1: Fig. S5).

### GSH- and pH-mediated release of Mn^2+^ and GAL

**Fig. 2:**
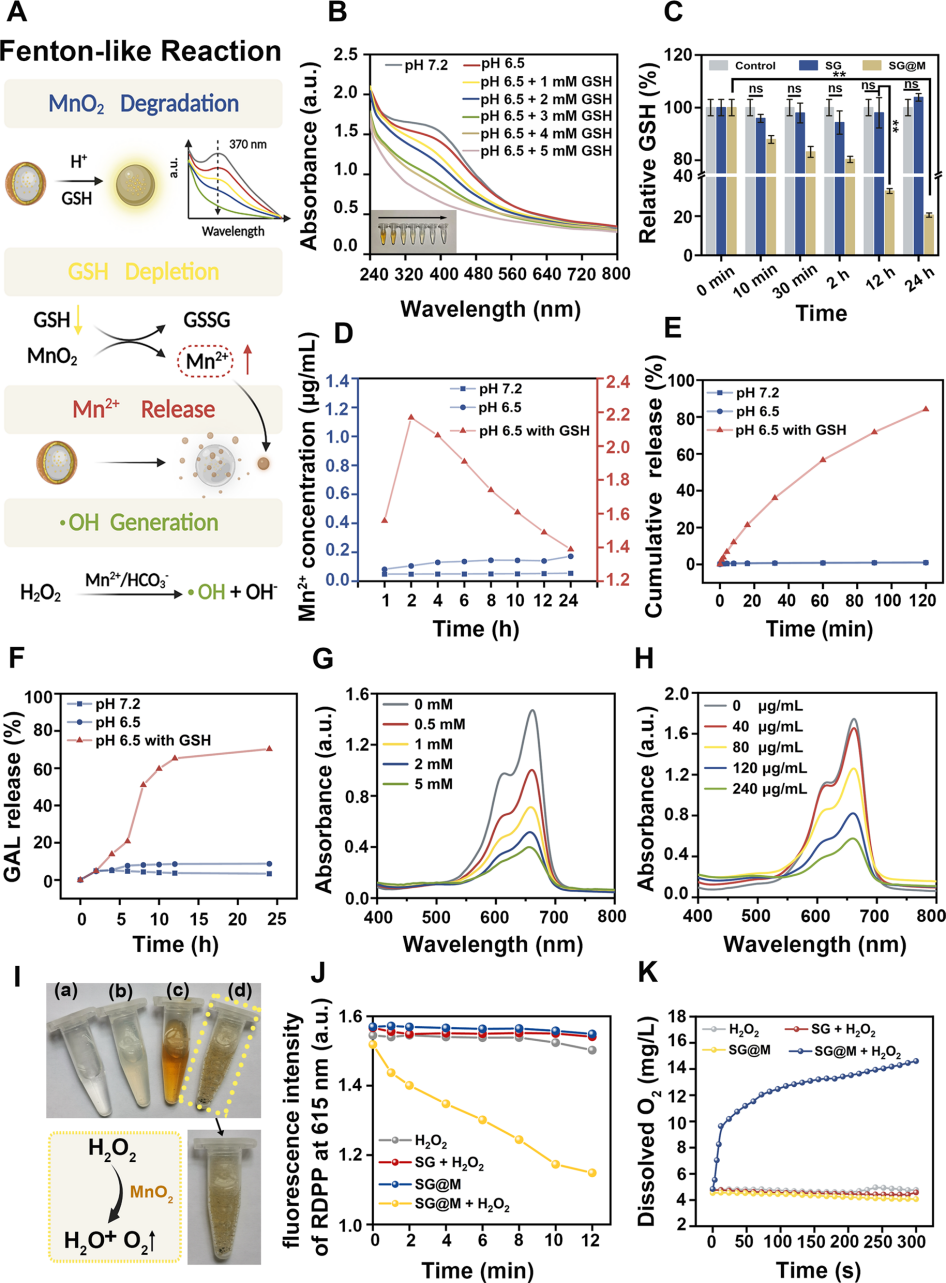
TME-triggered cascade effect of SG@M. **A** Schematic illustration of Fenton-like reaction (Created with BioRender.com). **B** UV–vis absorption spectra of the degradation properties of SG@M triggered by H^+^ and GSH and the corresponding photos of solution (inset). **C** Relative GSH content at different time points after the reaction with different NPs (n = 3). **D** Mn^2+^ release from SG@M at pH 7.2, pH 6.5 with or without GSH buffer solution after 24 h of incubation and **E** 2 h of incubation. **F** Drug release from SG@M at pH 7.2, pH 6.5 with or without GSH buffer solution after 24 h of incubation. **G** UV–vis absorption spectra of MB degradation in acid (pH 6.5) with different concentrations of GSH and **H** different concentrations of SG@M. **I** Photos of the reactions between (a) H_2_O_2_, (b) SG and H_2_O_2_, (c) SG@M, (d) SG@M and H_2_O_2_, respectively. **J** The O_2_ generation ability of SG@M by measuring RDPP fluorescence at 615 nm. **K** Dissolved O_2_ concentration in different solutions

The stepwise degradation of SG@M could induce controlled drug release and a Fenton-like reaction (Fig. 2A). Approximately 1−10 mM glutathione (GSH) exists in tumor cells [65]. A high concentration of GSH has strong antioxidant ability and can effectively scavenge ROS, which may restrict the efficacy of CDT [34, 38] and OS damage. To confirm the ability of the MnO_2_ shell to deplete GSH in the TME, we compared the variation in SG@M NPs in different buffers (pH 7.2 and pH 6.5) as well as different concentrations of GSH in the acidic buffer (pH 6.5). The progressive fading of the brown solution, which can be seen as the first sign of the reaction between MnO_2_ and GSH (inset in Fig. 2B), was later confirmed by the decrease in UV−Vis absorption at 370 nm with an increase in GSH content (Fig. 2B). We also found that a minor degradation of MnO_2_ occurred in an acidic environment (pH 6.5) compared to a normal environment (pH 7.2), but the degradation could be accelerated with the addition of GSH, which trend was not so pronounced in the solution at pH 7.2 (Additional file 1: Fig. S6), suggesting that H^+^ and GSH are co-conditions that trigger the degradation of MnO_2_. DTNB is commonly used as an indicator to measure GSH content due to the weakening of its characteristic UV–Vis absorption peak at 412 nm when reacting with GSH [38]. SG@M NPs consumed GSH in a time-dependent manner (Fig. 2C). The content of GSH decreased by 13.1% at 10 min and only remained at 20.5% at 24 h in the SG@M group; however, there was no variation in GSH content in the control group or the SG group.

Along with consuming GSH, the MnO_2_ shell also converts Mn^4+^ to Mn^2+^, which facilitates the subsequent process. The concentration of Mn^2+^ peaked at 2 h in an environment where GSH and H^+^ existed simultaneously (pH 6.5 with GSH), as shown in Fig. 2D, and then decreased when adding the external solution of the simulation system. While there was no discernible difference in the normal system (pH 7.2), the concentration of Mn^2+^ in the acidic system (pH 6.5 without GSH) slowly increased over time. Therefore, we further explored the cumulative release of Mn^2+^ over 2 h. More than 80% of Mn^2+^ was released from SG@M in the pH 6.5 solution containing GSH after 2 h of incubation, and GSH accelerated the release of Mn^2+^ (Fig. 2E). We also found that H_2_O_2_ in acidic conditions may be a factor causing the degradation of MnO_2_, which is due to the CAT-like properties of MnO_2_ (Additional file 1: Fig. S7). These results indicated that the MnO_2_ shell was degraded in the TME. Due to the CAT-like properties, acid sensitivity and GSH responsiveness, SG@M NPs can not only control the release of GAL but also perform CDT and MRI/USI activity simultaneously.

After the MnO_2_ shell completes its function, SG NPs are exposed and GAL is released. To investigate the release of GAL from SG@M NPs in an acidic and overexpressed GSH environment, we monitored the release of GAL by simulating the environments of normal and tumor tissues, respectively. Under pH 6.5 and 5 mM GSH conditions, the GAL release rate reached 70.3% at 24 h, while the release rate at pH 7.2 and pH 6.5 was only 3.3% and 8.7% at 24 h, respectively (Fig. 2F), which was due to the non-degradation of MnO_2_ in a normal environment and slow degradation in an acidic environment only, echoing the results of Mn^2+^ release. Additionally, the SiO_2_ nanospheres we prepared were loose and self-degradable. After SG NPs were dispersed in different solutions, we found that the content of Si in each solution gradually increase with time and the trend is basically the same, indicating that SiO_2_ carrier possesses the property of spontaneous decomposition (Additional file 1: Fig. S8). This is due to the fact that the special type of SiO_2_-drug composite NPs we prepared combines the self-release of drugs with the self-degradation of SiO_2_ carrier simultaneously, and the introduction of the drug GAL into SiO_2_ during the growth of SiO_2_ NP creates a radial concentration gradient of GAL, the release of which is mainly determined by diffusion, which in turn triggers the decomposition of the SiO_2_ carrier [37, 54, 59]. This can be considered as a safe carrier of drugs and an in-situ growth template of MnO_2_. However, compared with SG NPs, only a small amount of Si was released in the pH 7.2 group and pH 6.5 group in the SG@M NPs because of the protection of MnO_2_, and the Si content is gradually increased with the degradation of MnO_2_ in the pH 6.5 GSH-containing group (Additional file 1: Fig. S9), further demonstrating that MnO_2_ only degraded in the specific environment, which could prevent early drug leakage. The above results demonstrated that SG@M NPs only function in specific TME by gradual degradation and selective drug release; moreover, GSH would accelerate the degradation of SG@M.

MB is usually applied to indirectly reflect the content ·OH generated by Mn^2+^. The characteristic absorbance of MB at 665 nm decayed with increasing concentrations of GSH and SG@M NPs. (Fig. 2G, H and Additional file 1: Fig. S10). This could be explained by the fact that large amounts of Mn^2+^ were released from the increased SG@M after reaction with excessive GSH, mediating the Fenton-like reaction to generate substantial amounts of ·OH.

MnO_2_ targets H_2_O_2_ overexpressed in the TME, catalyzes its production of O_2_, and relieves hypoxia in a manner similar to catalase activity [41, 66–69]. Therefore, we monitored the ability of SG@M to produce O_2_ under different conditions. As shown in Fig. 2I (d), many bubbles emerged due to the simultaneous presence of MnO_2_ and H_2_O_2_, and to confirm that these bubbles were indeed made of O_2_, we used the O_2_-sensitive probe RDPP, whose fluorescence was suppressed by O_2_. When the fluorescence intensity at 615 nm was measured, it was discovered that the absorbance dropped immediately after the SG@M was incubated with H_2_O_2_ and persisted for 12 min, demonstrating that significant O_2_ was produced during this process. However, there was no decrease in the absorbance in the pure H_2_O_2_, SG@M, or SG with H_2_O_2_ groups (Fig. 2J). We then used a dissolved oxygen monitor to track the O_2_ concentration. Figure 2K showed the changes in O_2_ concentration in the four groups. In the H_2_O_2_ solution with SG@M added, the dissolved O_2_ rapidly increased from 4.8 mg/L to 10.1 mg/L and continued to increase compared to the previous three groups that lacked O_2_ trigger conditions. This further demonstrated the ability of SG@M to generate O_2_ to effectively alleviate hypoxia and for subsequent enhanced USI.

### Intracellular ROS production and a cascade of catalytic effect

**Fig. 3:**
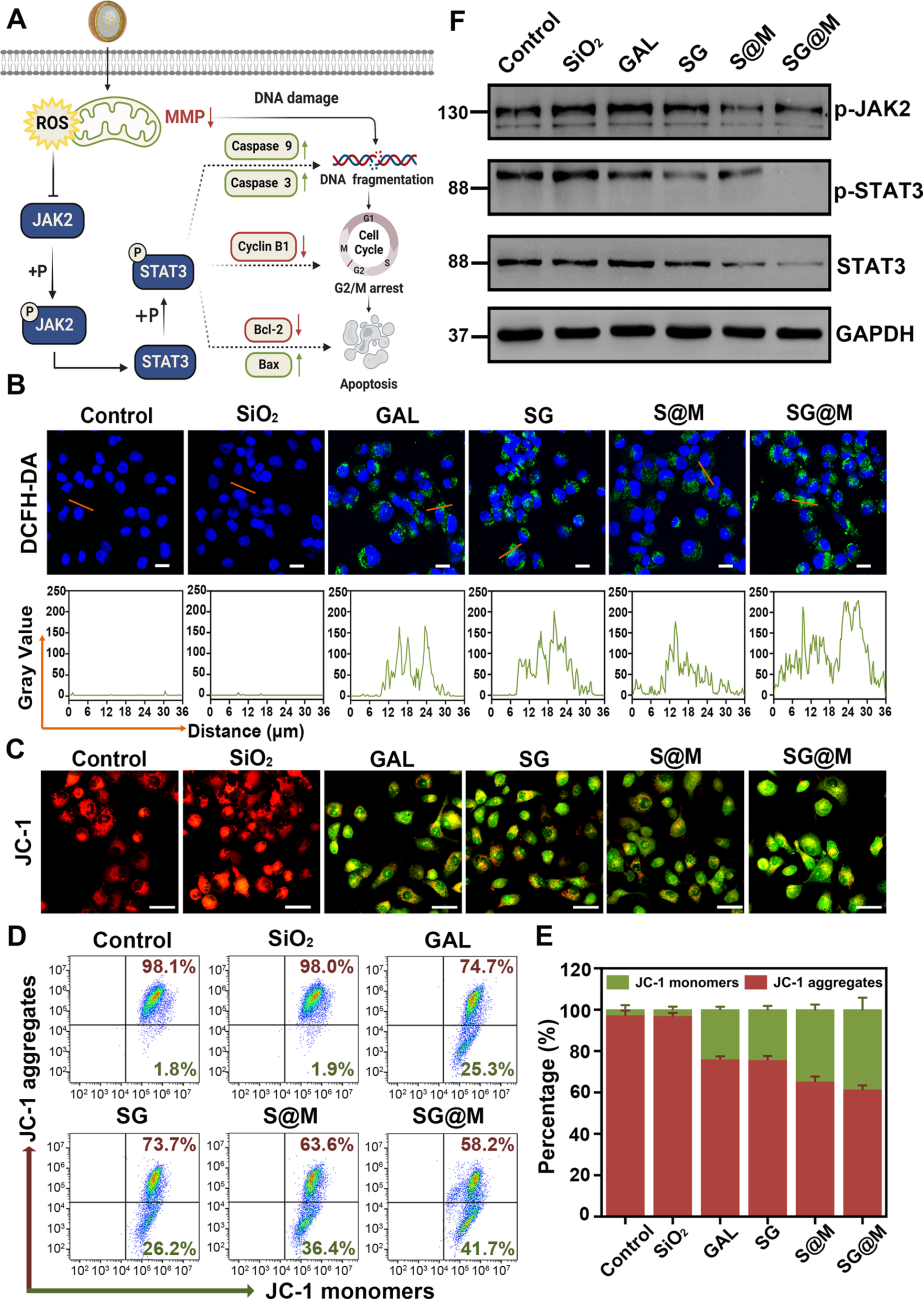
Intracellular ROS generation, MMP alteration and JAK2/STAT3 expression. **A** Schematic illustration of ROS causing cell apoptosis through multiple pathways (Created with www.BioRender.com). **B** LSCM images of intracellular ROS levels. Scale bar: 20 µm. **C** LSCM images of MMP changes after different treatments. Scale bar: 50 µm. **D** Flow cytometry analysis of MMP changes and **E** corresponding JC-1 aggregates/monomers percentages (n = 3). **F** Western blot analyses of phospho-JAK2 (Tyr 1007), STAT3, phospho-STAT3 (Tyr 705) protein levels in PANC-1 cells after different treatments

High quantities of ROS are harmful to cancer cells, resulting in DNA destruction as well as mitochondrial membrane disruption that releases and translocates cytochrome c into the cytoplasm, as seen by a decrease in MMP. Together with apoptosis protease-activating factor 1 (Apaf-1) and autoactivated Caspase 9, cytochrome c can create an apoptosome that will activate Caspase 3 and carry out the apoptotic process [70]. More importantly, ROS is one of the causes of cell cycle arrest [71] and one of the multiple cytokines that the JAK/STAT signaling pathway [71–74] responds to (Fig. 3A). Therefore, we explored the ability of SG@M to produce ROS in PANC-1 cells and the ROS-induced cascade effect. First, cellular uptake of SG@M was evaluated at different incubation times (0.5, 1, 2, 3 and 4 h). LSCM images revealed that cellular endocytosis grew as the incubation time increased, reaching its peak fluorescence intensity at 4 h, and the quantitative uptake of flow cytometry was 98% (Additional file 1: Fig. S11), indicating that SG@M was effectively endocytosed into the cells after 4 h of incubation. After the cells had been subjected to various treatments for 24 h, the ROS production was then monitored by staining with the ROS probe DCFH-DA. In contrast to the control group and SiO_2_ group, which did not exhibit any green fluorescence, the remaining four groups of free GAL, SG, S@M and SG@M showed mild green fluorescence, with the SG@M group exhibiting the strongest fluorescence intensity (Fig. 3B).

Intracellular ROS can cause mitochondrial malfunction, leading to alterations in membrane potential, and a drop in MMP reflected by the mitochondrial dye JC-1 also suggested the beginning of apoptosis [75]. The MMP was high and complete in the control and SiO_2_ groups, as shown in Fig. 3C, and showed strong red fluorescence. In contrast, the other four groups produced ROS that destroyed the intact MMP and exhibited enhanced green fluorescence with slight yellow fluorescence after merging. This was most noticeable in the SG@M group due to the high accumulation of ROS. To further quantify the changes in MMP, the ratios of the two forms of fluorescence were assessed using flow cytometry (Fig. 3D). While the percentages of green fluorescence increased in the other four groups, the control and SiO_2_ groups exhibited the highest red fluorescence percentages, which was consistent with the LSCM images. More importantly, the percentage of red fluorescence dropped by more than 40% in the SG@M group (Fig. 3E). Mitochondrial dysfunction results in mitochondrial outer membrane permeabilization and cytochrome c release, which activates Caspase 9, Caspase 3, and finally causes apoptosis [76]. Unsurprisingly, the SG@M group displayed the highest relative activity of Caspase 9 and Caspase 3 in accordance with the MMP results (Additional file 1: Figs. S12 and S13). According to the above results, SG@M may significantly increase ROS levels in cells and initiate apoptosis via the mitochondrial pathway, which is followed by anticancer effects.

Additionally, western blotting was utilized to examine how the JAK2/STAT3 signaling pathway in PANC-1 cells responded to ROS produced by the SG@M interaction. Figure 3F revealed that, after exposure to SG or S@M, a notable reduction in the levels of phospho-JAK2 and phospho-STAT3 was observed, while the strongest inhibitory effect was found in cells exposed to SG@M. According to these results, we could reasonably hypothesize that SG@M considerably increased the level of ROS, which might cause oxidative stress and possibly significantly alter many anticancer activities, including the regulation of the mitochondrial pathway and STAT3, and might facilitate tumor cell apoptosis.

### OS-related cytotoxicity study of SG@M in vitro

**Fig. 4:**
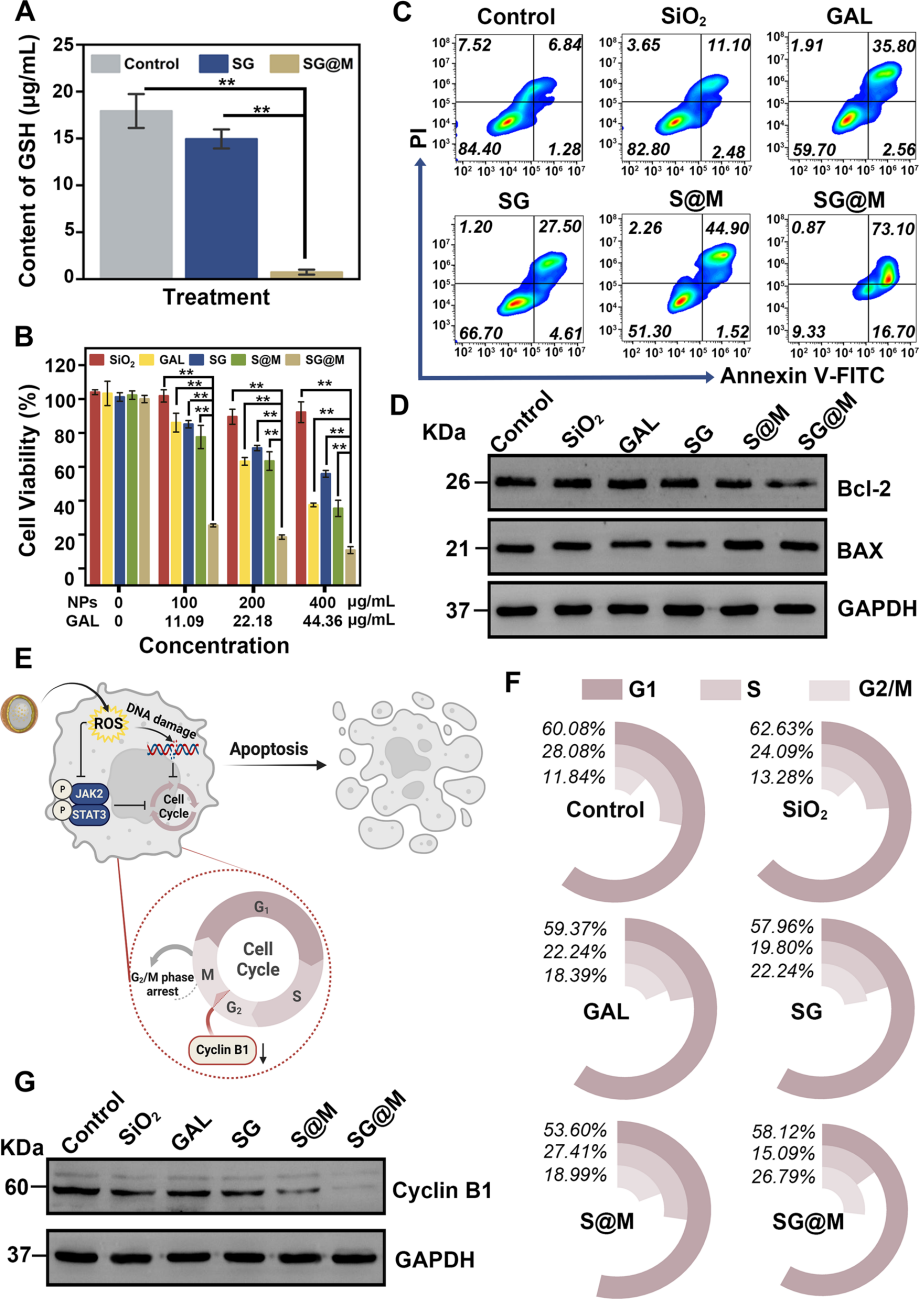
Antitumor therapy of SG@M in vitro. **A** Intracellular GSH level after different treatments (n = 3). **B** Cell viability of PANC‒1cells with different treatments (n = 3). **C** Flow cytometry analysis of cell apoptosis after different treatments (200 μg/mL SG or SG@M, equivalent to 22.18 μg/mL GAL). **D** Western blot analyses of BAX and Bcl-2 protein levels in PANC-1 cells after different treatments. **E** Molecular mechanism of cell growth arrest and apoptosis triggered by ROS (Created with www.BioRender.com). **F** Flow cytometry analysis of cell cycle distribution and **G** Western blot analyses of Cyclin B1 protein levels of PANC-1 cells in different treatment groups

ROS may be scavenged by GSH to maintain redox homeostasis, so we evaluated the ability of SG@M to deplete intracellular GSH using a GSH assay kit. As shown in Fig. 4A, after PANC-1 cells were coincubated with SG and SG@M for 24 h, the GSH levels in the SG@M group were extremely reduced, while those in the control and SG groups remained high. Furthermore, the GSH levels showed a concentration trend in SG@M (Additional file 1: Fig. S14). This result implied that SG@M could effectively disturb redox homeostasis and further amplify the damage caused by oxidative stress. Next, we investigated the anticancer efficacy of oxidative stress on PANC-1 cells. As expected, in Fig. 4B, the CCK-8 results showed that there was no decline in viability after cells were treated with SiO_2_, but small amounts of cell death occurred in the SG group carrying GAL due to the toxicity of GAL to cells. The CDT impact of Mn^2+^ in S@M may be able to inhibit the growth of tumor cells, as the cell viability rates dropped in the S@M group and showed a concentration and time trend (Additional file 1: Fig. S15). The lowest cell viability, 10.8%, was observed in cells treated with SG@M (400 µg/mL, equivalent to 44.36 µg/mL GAL) at the same concentration. In Fig. S16 of additional file 1, the combined therapeutic efficiency of SG@M was higher than the additive therapeutic efficiency of loading GAL and CDT alone, demonstrating the oxidative damage amplification efficacy of SG@M [62, 63]. Flow cytometry further confirmed the killing effect of SG@M, which had the highest apoptosis rate of 73.1% at a concentration of 200 µg/mL (Fig. 4C). In addition, western blotting analysis of the expression of proteins reflecting apoptosis in PANC-1 cells revealed that the upregulated BAX levels and the downregulated Bcl-2 levels were more significant after SG@M treatment (Fig. 4D), which is consistent with the findings of increasing apoptosis presented above.

An interesting observation was that the viability of cells exposed to high concentrations of SG (400 µg/mL, equal to 44.36 µg/mL GAL) was higher than that of free GAL, which was reasonably explained by the fact SiO_2_, as a sustained-release drug carrier, reduced the present concentration of GAL at the same time point, leading to a reduction in cytotoxicity compared to the same concentration of free GAL (Additional file 1: Fig. S17). This lays the foundation for the slow entry of the drug into the blood and the sustained long-term release to reduce or overcome the systemic toxicity or adverse effects caused by the administration of the drug alone [77–79]. To further verify such property of SiO_2_, we loaded two self-luminous drugs, i.e., MB and doxorubicin (DOX), with SiO_2_ and compared the intracellular fluorescence intensity at the same time point with LSCM and flow cytometry. Compared to the red fluorescent spots of concentrated MB observed in the free MB group, only occasional intense fluorescent spots were found in the SiO_2_-MB group, while a rather diffusive fluorescence background (Additional file 1: Fig. S18a, b), thus the intracellular fluorescence intensity was lower than in the free MB group (Additional file 1: Fig. S18c). This is attributed to the sustained escape of MB from SiO_2_ and its eventual release into the cytoplasm. Similar results (Additional file 1: Fig. S19) were obtained with cellular uptake of DOX or SiO_2_-DOX.

Increased intracellular ROS can cause DNA damage and decrease the levels of cyclin B1 [80], which is one of the causes of G2/M phase cell cycle arrest (Fig. 4E). To investigate whether SG@M-induced ROS production was associated with cell cycle arrest, we analyzed cell cycle changes after 24 h of different treatments by flow cytometry. The proportion of cells in the G2/M phase rose from 11.84% (control) to 18.39% (GAL), 22.24% (SG), 18.99% (S@M), and 26.79% (SG@M), as shown in Fig. 4F. When cells were exposed to GAL, SG, S@M, and SG@M, western blot analysis revealed that cyclin B1, a crucial element involved in the transition of the cell cycle from G2 to M phase, was downregulated (Fig. 4G). Accordingly, the in vitro results implied that SG@M exerted antitumor therapeutic effects on PANC-1 cells through oxidative DNA damage, growth arrest and cell death.

### USI/MRI capability and the metabolism of SG@M

**Fig. 5:**
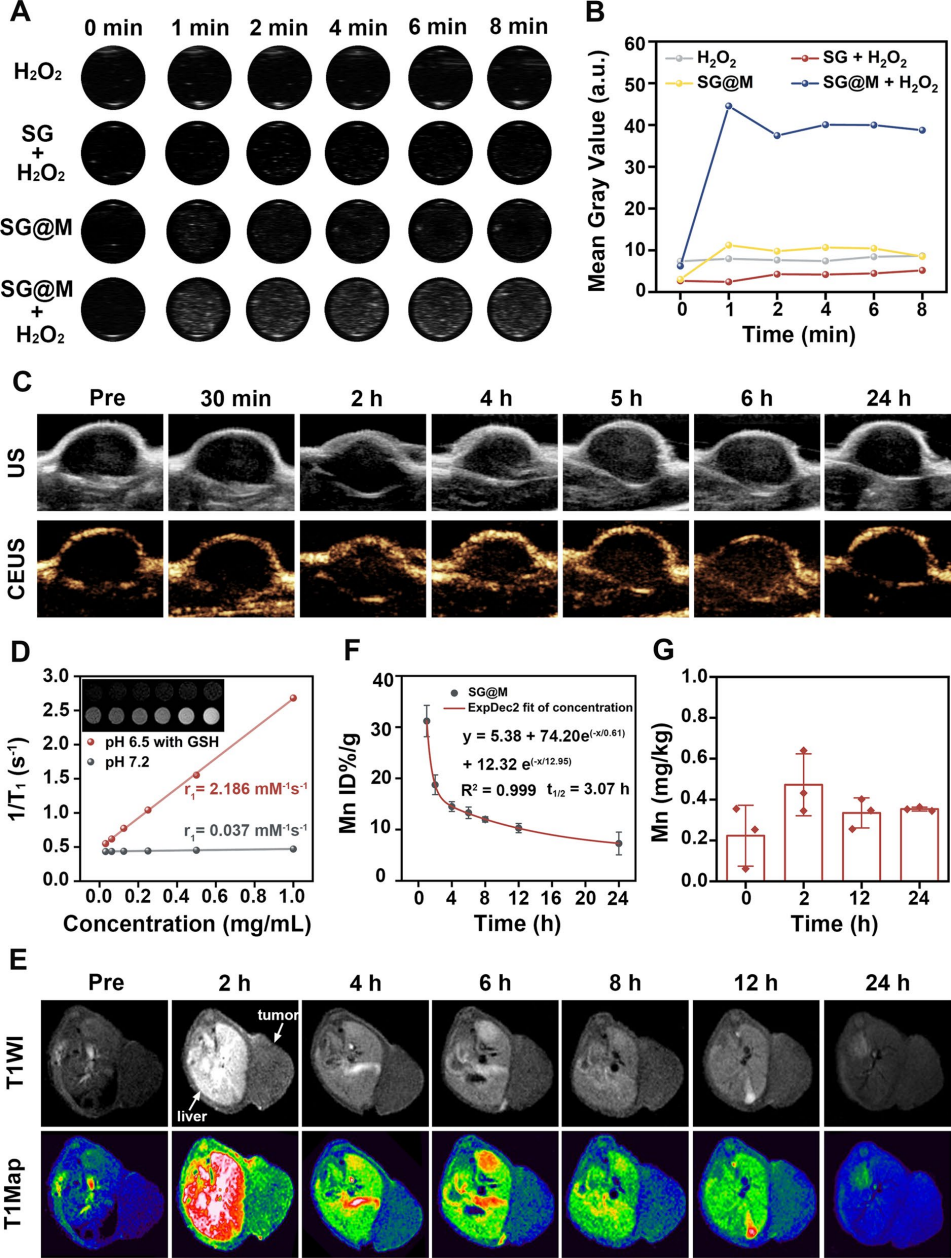
US and MR imaging in vitro and in vivo. **A** In vitro US images of H_2_O_2_, SG + H_2_O_2_, SG@M and SG@M + H_2_O_2_ at different time points and **B** corresponding mean gray values. **C** In vivo US and CEUS images of tumors before and after intravenous injection of SG@M at different time points. **D** T1-weighted MR images of different concentrations of SG@M in the pH 7.2 and pH 6.5 containing GSH (5 mM) buffer and the relative T1 relaxation rates. **E** T1-weighted MR images of tumor-bearing mice before and after intravenous injection of SG@M at different time points. **F** The blood circulation and **G** accumulation within tumor tissues for Mn in tumor-bearing mice administrated SG@M (n = 3)

Excess H_2_O_2_ in the TME is a prerequisite for SG@M-triggered O_2_ generation for enhanced US imaging. Considering the remarkable catalytic ability of SG@M for H_2_O_2_ in vitro, we further investigated the US imaging potential of SG@M with sustained O_2_ release capability. We initially measured the O_2_ production of SG@M in PANC-1 cells also using the RDPP probe, which exhibited intense red fluorescence under hypoxia and was gradually quenched after 12 h and 24 h of coincubation due to O_2_ production (Additional file 1: Fig. S20). We then explored O_2_-enhanced USI in vitro and in vivo. The US signal strength clearly increased when SG@M and H_2_O_2_ were present simultaneously, as shown in Fig. 5A and B, but H_2_O_2_ alone, SG@M alone, and without the addition of MnO_2_ (SG + H_2_O_2_) did not demonstrate any discernible changes. To confirm the US imaging ability in vivo, a mouse model of pancreatic tumors was utilized to observe the signal changes within 24 h after *i.v.* injection of SG@M. Figure 5C showed a low US signal in the tumor region before the injection of SG@M. Subsequently, the signal intensity tended to be brighter with prolonged injection; it peaked at 6 h and subsided at 24 h, indicating the effective aggregation and metabolism of SG@M at the tumor site. The contrast-enhanced ultrasound (CEUS) mode also displayed the same trend. The increased signal intensity was mainly due to SG@M that exerted CAT-like activity in the tumor region to continuously produce O_2_ bubbles and generate sufficient echogenic reflectivity to reinforce US signal. Additionally, by utilizing enhanced permeability and retention (EPR) effect-mediated passive, progressive, and relatively selective accumulation at the diseased location over time, CEUS imaging can be used to describe malignancies.

In addition to the properties of SG@M for US imaging, the Mn^2+^ produced by its degradation guided T1-weighted MRI. As usual, we first demonstrated its MRI performance in vitro. Compared with the normal system (pH 7.2), most Mn^2+^ was released in the acidic (pH 6.5) containing GSH system, which showed the strongest T1 signal (Fig. 5D). As the concentration of SG@M increased, the images also became brighter and had the longest longitudinal relaxation rate (r_1_ = 2.186 mM^−1^ s^−1^), indicating that SG@M has significant MRI properties. Then we investigated whether SG@M could exhibit this performance in vivo in tumor-bearing mice. Figure 5E exhibited that after *i.v.* injection of SG@M, the tumor location became brighter over time, peaking at 2 h. Interestingly, the variations in signal strength at the liver were identical to those seen in tumors; they peaked at 2 h and remained for a long period, and the signal was still visible at 24 h. This suggested that SG@M not only functioned by aggregating at the tumor site with blood but was also metabolized out of the body through the liver system over time, thus minimizing the side effects on normal organs [38].

To examine the metabolism of SG@M, the Mn^2+^ content of the tumor and blood circulation was detected using ICP-MS to determine the metabolism of SG@M. As shown in Fig. 5F, the circulating half-life was calculated to be 3.07 h, which is necessary for the effective penetration of nanospheres into tumors through the EPR effect and their subsequent metabolization. The analysis of Mn^2+^ content in tumors showed that SG@M accumulated in the tumor site at 2 h after injection, and although it slightly decreased at 12 h, it continued to be higher than before administration until 24 h (Fig. 5G). This corresponded to the in vivo MRI results, indicating that SG@M was successfully degraded in the TME and generated Mn^2+^ sufficiently to enhance MRI. However, the signal value of in vivo USI peaked at 6 h, later than MRI, probably because O_2_ production catalyzed by MnO_2_ was a slow process. When SG@M arrived at the tumor site, the shell MnO_2_ was first rapidly degraded to Mn^2+^ to enhance MRI, thus only a small amount of O_2_ was produced. More SG@M accumulated in the tumor over time, and the excess MnO_2_ catalyzed the continuous release of O_2_ bubbles to function as a US reflector, resulting in sufficient echogenic reflectivity, highly favoring US imaging. Based on these experimental results, it could be confirmed that SG@M can be considered a promising dual-modal imaging contrast agent for precise diagnosis, real-time tracking, continuous monitoring, and US/MR imaging-guided delivery.

### OS-related antitumor therapy of SG@M in vivo

**Fig. 6:**
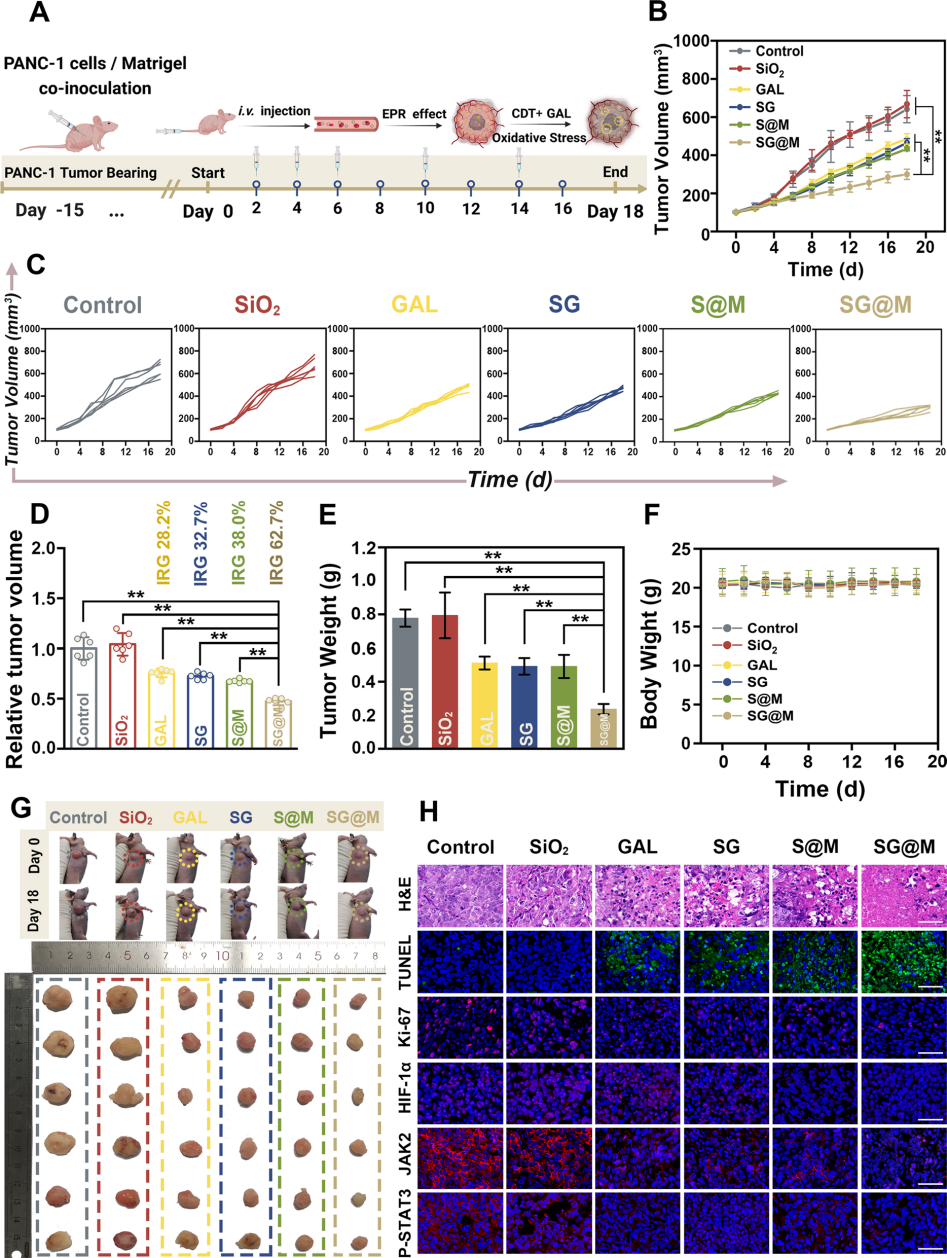
In vivo ROS-related therapy of SG@M. **A** The schematic illustration of mice tumor therapy (Created with BioRender.com). **B** Tumor growth curves and **C** tumor volume changes in six groups during treatment (n = 6). **D** The relative tumor volume and inhibition rate of tumor growth (IRG) and **E** the tumor weight at the end of treatment (n = 6). **F** The bodyweight during treatment (n = 6). **G** Photos of mice before and after 18 days of treatment and corresponding tumors dissected from mice in six groups after 18 days of treatment. **H** H&E/TUNEL/Ki-67/HIF-1α/JAK2/p-STAT3 staining of tumor sections after 18 days of treatment in six groups. Scale bar: 50 µm

Motivated by the effective ROS-related anticancer activity of SG@M in vitro and the significant tumor accumulation of SG@M in vivo, we explored the oxidative stress-mediated antitumor performance of SG@M in vivo with PANC-1 tumor-bearing mice. As seen in Fig. 6A, a total of five injections of different formulations, including saline, SiO_2_, free GAL, SG, S@M, and SG@M, were administered during the 18-day observation period. As indicated in the tumor growth curves (Fig. 6B, C), the control and SiO_2_ groups exhibited faster tumor growth, while the combined group (SG@M) had the most pronounced inhibitory effect on tumor progression. On the 18th day, the inhibition rate of tumor growth (IRG) [81] was 28.2%, 32.7% and 38.0% in the GAL, SG and S@M groups, respectively (Fig. 6D), and the combination treatment group (SG@M) exhibited the most potent tumor inhibition, with an IRG of 62.7%. This was attributable to the synergistic impact of SG@M, which increased oxidative stress while also acting as an anticancer agent. In addition, photos of tumors on the 18th day (Fig. 6G) and tumor weights in vitro (Fig. 6E) showed similar trends, without a significant weight decrease in mice during the observation period (Fig. 6F). Following the observation period of treatment, tumor tissues from each group were extracted and examined for hematoxylin and eosin (H&E), Ki-67, and TUNEL immunofluorescence (IF) staining (Fig. 6H). The H&E-stained tumor slices from the SG@M group displayed significant areas of necrosis by presenting the tumor cells, in contrast to other groups. This corresponded to the results of the TUNEL analysis, which showed stronger green fluorescence. Additionally, the Ki-67 marker was applied to label the proliferating cells in various groups. Undoubtedly, the red fluorescence of Ki-67 in the SG@M group was less than that in the other groups, indicating that the combined effect of SG@M amplified oxidative stress and effectively inhibited cancer cell proliferation.

In view of the exceptional O_2_ production capacity of SG@M, IF staining of tumor slices for hypoxia-inducible factor-1α (HIF-1α) was performed to verify whether SG@M could alleviate hypoxia in vivo. Figure 6H revealed that except for the S@M and SG@M groups with the effect of MnO_2_, the pre four groups showed strong red IF signals accompanied by overexpression of HIF-1α, indicating serious hypoxic levels inside the tumors. The faint red fluorescent signal of HIF-1α revealed that its expression level was drastically downregulated in the final two groups, as predicted. Such a reduction in HIF-1α was attributed to the CAT-like effect of MnO_2_, suggesting the role of O_2_ production from SG@M in alleviating tumor hypoxia. Similarly, subject to the JAK2/STAT3-mediated tumor suppressive effect of SG@M in vitro, we explored the effect of SG@M on this pathway in vivo. As seen by the IF results of JAK2 and p-STAT3 from tumor slices, SG@M inhibited this signaling pathway (Fig. 6H), which was consistent with their effect on PANC-1 cells. Taken together, these cascade effects prevent the development and metastasis of PANC-1 tumors.

### Biocompatibility of SG@M

**Fig. 7:**
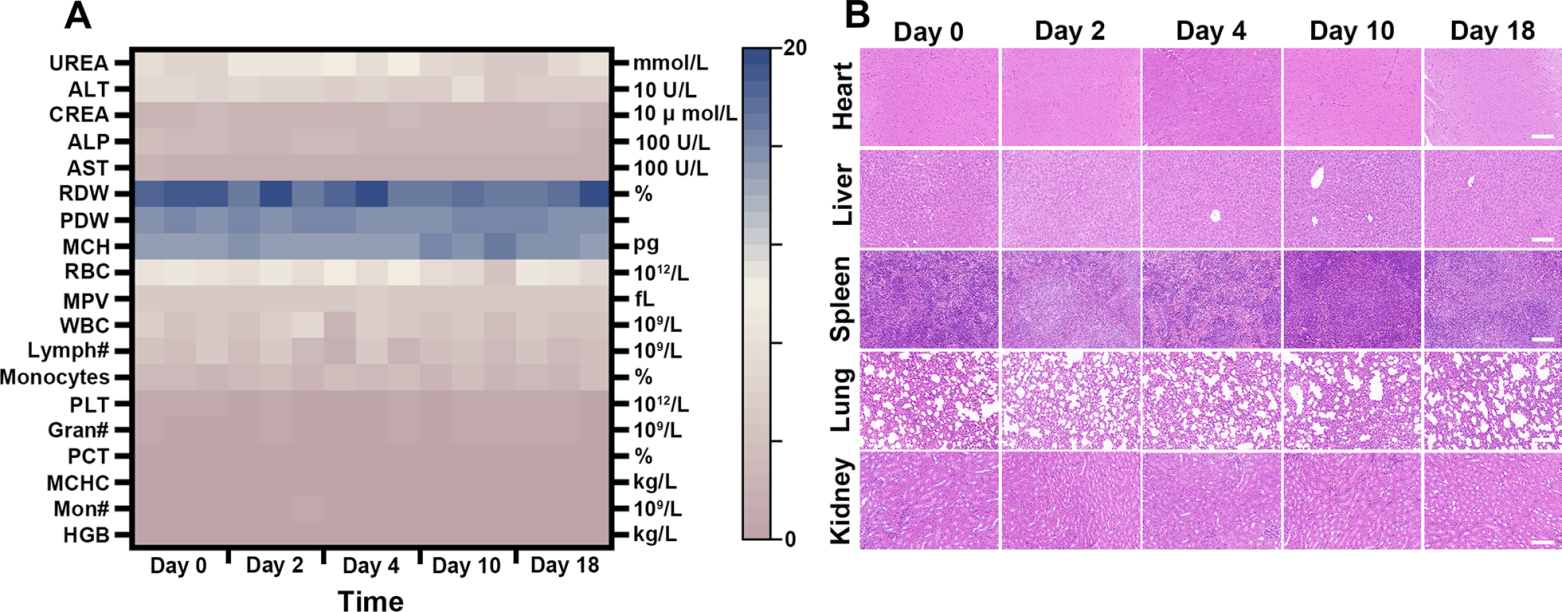
Biocompatibility evaluation of SG@M. **A** Analysis of biochemical parameters in mice (n = 3) at different times of administration of SG@M. **B** H&E staining of the major organs at corresponding time intervals. Scale bar: 100 µm

Finally, we investigated the biosafety of SG@M in vivo during the observation period. After short- or long-term SG@M injection, liver function, renal function, and other blood indices revealed no abnormalities (Fig. 7A). H&E staining of the major organs in mice also showed no changes after injection (Fig. 7B), indicating that SG@M exhibited a favorable biosafety profile.

## Conclusion

In this study, we successfully loaded GAL into S@M nanoagents with CDT characteristic to form SG@M by a two-step approach. This hybrid nanopharmaceutical could not only respond to the TME that allows spontaneous cascade reactions, but also effectively control the release of encapsulated GAL. On the one hand, it mediates the Fenton-like reaction that stimulates intracellular ROS levels together with the released GAL while depleting GSH to weaken ADS, disrupting the intrinsic redox homeostasis in tumors and further regulating oxidative stress levels, and also synergistically inhibits the JAK2/STAT3 pathway to suppress the cell proliferation process in the G2/M phase. On the other hand, the O_2_ that alleviates tumor hypoxia improves USI, and Mn^2+^ that plays a role in CDT improves MRI, proving that “the therapy is simple but the efficacy is profound” and providing a novel notion for oxidative stress therapy to inhibit the progression of pancreatic cancer or other malignant cancers.

## Supplementary Information

**Additional file 1:** **S1. Methods**. **Figure S1. **Size and Zeta potential of SG and SG@M (n = 3). **Figure S2.** XPS analysis results of SG@M, and the enlarged elements patterns of O, C and Si. **Figure S3. **UV−vis absorption spectrum of SG and SG@M and photos of the corresponding solutions (inset). **Figure S4. **Structural formula of galangal (GAL). **Figure S5. **Standard curve of GAL based on HPLC and chromatogram of GAL content in SG@M. (n = 3). **Figure S6.** UV-vis absorption spectra of SG@M in PBS (pH=7.2) containing different concentrations of GSH. **Figure S7. **Mn^2+^ release from SG@M in various systems. **Figure S8. **Degraded Si amount from SG in different systems. **Figure S9. **Degraded Si amount from SG@M in different systems. **Figure S10. **The trend of absorbance of MB at 665 nm with SG@M and GSH concentration. **Figure S11. **LSCM images of cellular uptake of SG@M and corresponding flow cytometry analysis. Scale bar: 50 µm. (DiI labeled SG@M NPs and DAPI labeled cell nuclei). **Figure S12. **Standard curve of* ρ*NA and relative Caspase 9 activity in various treatment groups. (n = 3). **Figure S13.** Standard curve of* ρ*NA and relative Caspase 3 activity in various treatment groups. (n = 3). **Figure S14. **Standard Curve of GSH and the concentration trend of SG@M in cellular GSH levels (n = 3). **Figure S15. **The concentration and time trend in CDT effect of S@M (n=3). **Figure S16.** Therapeutic efficiency of synergistic effect (SG@M) compared with SG and CDT (S@M). The additive therapeutic efficiencies of independent SG and CDT treatments were estimated using the relation *T*_*additive*_ = 100 − (*f*_*SG*_ × *f*_*CDT*_) × 100, where *f* is the fraction of surviving cells after each treatment (n=3). **Figure S17.** The amount of GAL after incubating the cells with free GAL or SG NPs for 4 h and 24 h, respectively (n=3). **Figure S18. **The uptake levels of free MB and SiO_2_-MB. (a) LSCM images of PANC-1 cells treated with free MB or SiO_2_-MB (red fluorescence from MB, blue fluorescence from DAPI-labeled nuclei. Scale bars: 100 µm) and (c) corresponding transmittance images the morphologies of the specific cells (Scale bar: 50 µm). (c) Corresponding flow cytometry analysis (n=3). **Figure S19. **The uptake levels of free DOX and SiO_2_-DOX. (a) LSCM images of PANC-1 cells treated with free DOX or SiO_2_-DOX (red fluorescence from DOX, blue fluorescence from DAPI-labeled nuclei. Scale bars: 100 µm) and (c) corresponding transmittance images the morphologies of the specific cells (Scale bar: 50 µm). (c) Corresponding flow cytometry analysis (n=3). **Figure S20.** LSCM images of O_2_ production detected with RDPP after 12 h and 24 h incubation with SG@M. Scale bars: 50 µm.

## Data Availability

The data are available in the main manuscript, Additional information files are available from the corresponding author by request.

## References

[1] Sung H, Ferlay J, Siegel RL, Laversanne M, Soerjomataram I, Jemal A (2021). Global cancer statistics 2020: GLOBOCAN estimates of incidence and mortality worldwide for 36 cancers in 185 countries. CA Cancer J Clin.

[2] Stathis A, Moore MJ (2010). Advanced pancreatic carcinoma: current treatment and future challenges. Nat Rev Clin Oncol.

[3] Lei Y, Tang L, Xie Y, Xianyu Y, Zhang L, Wang P (2017). Gold nanoclusters-assisted delivery of NGF siRNA for effective treatment of pancreatic cancer. Nat Commun.

[4] Ferlay J, Partensky C, Bray F (2016). More deaths from pancreatic cancer than breast cancer in the EU by 2017. Acta Oncol.

[5] Sies H, Jones DP (2020). Reactive oxygen species (ROS) as pleiotropic physiological signalling agents. Nat Rev Mol Cell Bio.

[6] Martinez-Reyes I, Chandel NS (2021). Cancer metabolism: looking forward. Nat Rev Cancer.

[7] Cheung EC, Vousden KH (2022). The role of ROS in tumour development and progression. Nat Rev Cancer.

[8] Gorrini C, Harris IS, Mak TW (2013). Modulation of oxidative stress as an anticancer strategy. Nat Rev Drug Discov.

[9] Harris IS, DeNicola GM (2020). The complex interplay between antioxidants and ROS in cancer. Trends Cell Biol.

[10] Hayes JD, Dinkova-Kostova AT, Tew KD (2020). Oxidative stress in cancer. Cancer Cell.

[11] Martindale JL, Holbrook NJ (2002). Cellular response to oxidative stress: signaling for suicide and survival. J Cell Physiol.

[12] Wang Y, Qi H, Liu Y, Duan C, Liu X, Xia T (2021). The double-edged roles of ROS in cancer prevention and therapy. Theranostics.

[13] Dong Z, Feng L, Chao Y, Hao Y, Chen M, Gong F (2019). Amplification of tumor oxidative stresses with liposomal fenton catalyst and glutathione inhibitor for enhanced cancer chemotherapy and radiotherapy. Nano Lett.

[14] Harris IS, Endress JE, Coloff JL, Selfors LM, McBrayer SK, Rosenbluth JM (2019). Deubiquitinases maintain protein homeostasis and survival of cancer cells upon glutathione depletion. Cell Metab.

[15] Liu D, You P, Luo Y, Yang M, Liu Y (2018). Galangin induces apoptosis in MCF-7 human breast cancer cells through mitochondrial pathway and phosphatidylinositol 3-kinase/Akt inhibition. Pharmacology.

[16] Huang H, Chen AY, Ye X, Guan R, Rankin GO, Chen YC (2020). Galangin, a flavonoid from lesser galangal, induced apoptosis via p53-dependent pathway in ovarian cancer cells. Molecules.

[17] Liang X, Wang P, Yang C, Huang F, Wu H, Shi H (2021). Galangin inhibits gastric cancer growth through enhancing STAT3 mediated ROS production. Front Pharmacol.

[18] Zhong X, Huang S, Liu D, Jiang Z, Jin Q, Li C (2020). Galangin promotes cell apoptosis through suppression of H19 expression in hepatocellular carcinoma cells. Cancer Med.

[19] Arif H, Sohail A, Farhan M, Rehman AA, Ahmad A, Hadi SM (2018). Flavonoids-induced redox cycling of copper ions leads to generation of reactive oxygen species: a potential role in cancer chemoprevention. Int J Biol Macromol.

[20] Wang Y, Lin B, Li H, Lan L, Yu H, Wu S (2017). Galangin suppresses hepatocellular carcinoma cell proliferation by reversing the Warburg effect. Biomed Pharmacother.

[21] Kagawa TF, Geierstanger BH, Wang AH, Ho PS (1991). Covalent modification of guanine bases in double-stranded DNA. The 1.2-A Z-DNA structure of d(CGCGCG) in the presence of CuCl2. J Biol Chem.

[22] Ullah MF, Ahmad A, Khan HY, Zubair H, Sarkar FH, Hadi SM (2013). The prooxidant action of dietary antioxidants leading to cellular DNA breakage and anticancer effects: implications for chemotherapeutic action against cancer. Cell Biochem Biophys.

[23] Fang D, Xiong Z, Xu J, Yin J, Luo R (2019). Chemopreventive mechanisms of galangin against hepatocellular carcinoma: a review. Biomed Pharmacother.

[24] Lagoa R, Silva J, Rodrigues JR, Bishayee A (2020). Advances in phytochemical delivery systems for improved anticancer activity. Biotechnol Adv.

[25] Teng H, Zheng Y, Cao H, Huang Q, Xiao J, Chen L (2021). Enhancement of bioavailability and bioactivity of diet-derived flavonoids by application of nanotechnology: a review. Crit Rev Food Sci Nutr.

[26] Guo Y, Sun Q, Wu FG, Dai Y, Chen X (2021). Polyphenol-containing nanoparticles: synthesis, properties, and therapeutic delivery. Adv Mater.

[27] Chu PY, Tsai SC, Ko HY, Wu CC, Lin YH (2019). Co-delivery of natural compounds with a dual-targeted nanoparticle delivery system for improving synergistic therapy in an orthotopic tumor model. ACS Appl Mater Interfaces.

[28] Aiello P, Consalvi S, Poce G, Raguzzini A, Toti E, Palmery M (2021). Dietary flavonoids: nano delivery and nanoparticles for cancer therapy. Semin Cancer Biol.

[29] Zhang Z, Sang W, Xie L, Li W, Li B, Li J (2021). Polyphenol-based nanomedicine evokes immune activation for combination cancer treatment. Angew Chem Int Ed Engl.

[30] Yan J, Wang G, Xie L, Tian H, Li J, Li B (2022). Engineering radiosensitizer-based metal-phenolic networks potentiate STING pathway activation for advanced radiotherapy. Adv Mater.

[31] Sang W (2022). A triple-kill strategy for tumor eradication reinforced by metal-phenolic network nanopumps. Adv Funct Mater.

[32] Pezzani R, Salehi B, Vitalini S, Iriti M, Zuniga FA, Sharifi-Rad J (2019). Synergistic effects of plant derivatives and conventional chemotherapeutic agents: an update on the cancer perspective. Medicina (Kaunas).

[33] Hung SY, Lin SC, Wang S, Chang TJ, Tung YT, Lin CC (2021). Bavachinin induces G2/M cell cycle arrest and apoptosis via the ATM/ATR signaling pathway in human small cell lung cancer and shows an antitumor effect in the xenograft model. J Agric Food Chem.

[34] Lin LS, Song JB, Song L, Ke KM, Liu YJ, Zhou ZJ (2018). Simultaneous fenton-like ion delivery and glutathione depletion by MnO2-based nanoagent to enhance chemodynamic therapy. Angew Chem Int Edit.

[35] Yan N, Lin L, Xu C, Tian H, Chen X (2019). A GSH-gated DNA nanodevice for tumor-specific signal amplification of microRNA and MR imaging-guided theranostics. Small.

[36] Lyu M, Zhu D, Kong X, Yang Y, Ding S, Zhou Y (2020). Glutathione-depleting nanoenzyme and glucose oxidase combination for hypoxia modulation and radiotherapy enhancement. Adv Healthc Mater.

[37] He T, Jiang C, He J, Zhang Y, He G, Wu J (2021). Manganese-dioxide-coating-instructed plasmonic modulation of gold nanorods for activatable duplex-imaging-guided NIR-II photothermal-chemodynamic therapy. Adv Mater.

[38] Liu J, Zhang J, Song K, Du J, Wang X, Liu J (2021). Tumor microenvironment modulation platform based on composite biodegradable bismuth-manganese radiosensitizer for inhibiting radioresistant hypoxic tumors. Small.

[39] Ming L, Song L, Xu J, Wang R, Shi J, Chen M (2021). Smart manganese dioxide-based lanthanide nanoprobes for triple-negative breast cancer precise gene synergistic chemodynamic therapy. ACS Appl Mater Interfaces.

[40] Shen Z, Xia J, Ma Q, Zhu W, Gao Z, Han S (2020). Tumor Microenvironment-triggered Nanosystems as dual-relief tumor hypoxia immunomodulators for enhanced phototherapy. Theranostics.

[41] Zheng ZL, Jia Z, Qu CR, Dai R, Qin YF, Rong S (2021). Biodegradable silica-based nanotheranostics for precise MRI/NIR-II fluorescence imaging and self-reinforcing antitumor therapy. Small.

[42] Ding B, Zheng P, Ma P, Lin J (2020). Manganese oxide nanomaterials: synthesis, properties, and theranostic applications. Adv Mater.

[43] Yang G, Ji J, Liu Z (2021). Multifunctional MnO2 nanoparticles for tumor microenvironment modulation and cancer therapy. Wiley Interdiscip Rev Nanomed Nanobiotechnol.

[44] Wang Y, Li Y, Zhang Z, Wang L, Wang D, Tang BZ (2021). Triple-jump photodynamic theranostics: MnO2 combined upconversion nanoplatforms involving a type-I photosensitizer with aggregation-induced emission characteristics for potent cancer treatment. Adv Mater.

[45] Yang B, Chen Y, Shi J (2019). Nanocatalytic medicine. Adv Mater.

[46] Tang Z, Liu Y, He M, Bu W (2019). Chemodynamic therapy: tumour microenvironment-mediated fenton and fenton-like reactions. Angew Chem Int Ed Engl.

[47] Xiang H, You C, Liu W, Wang D, Chen Y, Dong C (2021). Chemotherapy-enabled/augmented cascade catalytic tumor-oxidative nanotherapy. Biomaterials.

[48] Xiang Q, Yang C, Luo Y, Liu F, Zheng J, Liu W (2022). Near-infrared II nanoadjuvant-mediated chemodynamic, photodynamic, and photothermal therapy combines immunogenic cell death with PD-L1 blockade to enhance antitumor immunity. Small.

[49] Zheng R, Cheng Y, Qi F, Wu Y, Han X, Yan J (2021). Biodegradable copper-based nanoparticles augmented chemodynamic therapy through deep penetration and suppressing antioxidant activity in tumors. Adv Healthc Mater.

[50] Feng Y, Ding D, Sun W, Qiu Y, Luo L, Shi T (2019). Magnetic manganese oxide sweetgum-ball nanospheres with large mesopores regulate tumor microenvironments for enhanced tumor nanotheranostics. ACS Appl Mater Interfaces.

[51] Wu F, Zhang Q, Sun B, Chu X, Zhang M, She Z (2021). MoO3-x nanosheets-based platform for single NIR laser induced efficient PDT/PTT of cancer. J Control Release.

[52] Chen H, Zheng D, Pan W, Li X, Lv B, Gu W (2021). Biomimetic nanotheranostics camouflaged with cancer cell membranes integrating persistent oxygen supply and homotypic targeting for hypoxic tumor elimination. ACS Appl Mater Interfaces.

[53] Tang L, Fan TM, Borst LB, Cheng J (2012). Synthesis and biological response of size-specific, monodisperse drug-silica nanoconjugates. ACS Nano.

[54] Zhang S, Chu Z, Yin C, Zhang C, Lin G, Li Q (2013). Controllable drug release and simultaneously carrier decomposition of SiO2-drug composite nanoparticles. J Am Chem Soc.

[55] Fukuda A, Wang SC, Morris JP, Folias AE, Liou A, Kim GE (2011). Stat3 and MMP7 contribute to pancreatic ductal adenocarcinoma initiation and progression. Cancer Cell.

[56] Corcoran RB, Contino G, Deshpande V, Tzatsos A, Conrad C, Benes CH (2011). STAT3 plays a critical role in KRAS-induced pancreatic tumorigenesis. Cancer Res.

[57] Baumgart S, Chen NM, Siveke JT, Konig A, Zhang JS, Singh SK (2014). Inflammation-induced NFATc1-STAT3 transcription complex promotes pancreatic cancer initiation by KrasG12D. Cancer Discov.

[58] Huang X, Dai S, Dai J, Xiao Y, Bai Y, Chen B (2015). Luteolin decreases invasiveness, deactivates STAT3 signaling, and reverses interleukin-6 induced epithelial-mesenchymal transition and matrix metalloproteinase secretion of pancreatic cancer cells. Onco Targets Ther.

[59] Ma Z, Jia X, Bai J, Ruan Y, Wang C, Li J (2017). MnO2 gatekeeper: an intelligent and O2-evolving shell for preventing premature release of high cargo payload core, overcoming tumor hypoxia, and acidic H2O2-sensitive MRI. Adv Func Mater.

[60] Wang D, Xue B, Ohulchanskyy TY, Liu Y, Yakovliev A, Ziniuk R (2020). Inhibiting tumor oxygen metabolism and simultaneously generating oxygen by intelligent upconversion nanotherapeutics for enhanced photodynamic therapy. Biomaterials.

[61] Fan WP, Bu WB, Shen B, He QJ, Cui ZW, Liu YY (2015). Intelligent MnO2 nanosheets anchored with upconversion nanoprobes for concurrent pH-/H2O2-responsive UCL imaging and oxygen-elevated synergetic therapy. Adv Mater.

[62] Park H, Yang J, Lee J, Haam S, Choi IH, Yoo KH (2009). Multifunctional nanoparticles for combined doxorubicin and photothermal treatments. ACS Nano.

[63] Wang X, Li C, Qian J, Lv X, Li H, Zou J (2021). NIR-II responsive hollow magnetite nanoclusters for targeted magnetic resonance imaging-guided photothermal/chemo-therapy and chemodynamic therapy. Small.

[64] Zhang M, Cushing BL, O'Connor CJ (2008). Synthesis and characterization of monodisperse ultra-thin silica-coated magnetic nanoparticles. Nanotechnology.

[65] Kosower NS, Kosower EM (1978). The glutathione status of cells. Int Rev Cytol.

[66] Liu XL, Dong X, Yang SC, Lai X, Liu HJ, Gao YH (2021). Biomimetic liposomal nanoplatinum for targeted cancer chemophototherapy. Adv Sci.

[67] Su Y, Zhang X, Lei L, Liu B, Wu S, Shen J (2021). Tumor microenvironment-activatable cyclic cascade reaction to reinforce multimodal combination therapy by destroying the extracellular matrix. ACS Appl Mater Interfaces.

[68] Wang Z, Liu B, Sun Q, Dong S, Kuang Y, Dong Y (2020). Fusiform-like copper(II)-based metal-organic framework through relief hypoxia and GSH-depletion Co-enhanced starvation and chemodynamic synergetic cancer therapy. ACS Appl Mater Interfaces.

[69] Yang J, Li K, Li CZ, Gu JL (2021). In situ coupling of catalytic centers into artificial substrate mesochannels as super-active metalloenzyme mimics. Small.

[70] Moloney JN, Cotter TG (2018). ROS signalling in the biology of cancer. Semin Cell Dev Biol.

[71] Li G, Fang S, Shao X, Li Y, Tong Q, Kong B (2021). Curcumin reverses NNMT-induced 5-fluorouracil resistance via increasing ROS and cell cycle arrest in colorectal cancer cells. Biomolecules.

[72] Bharadwaj U, Kasembeli MM, Robinson P, Tweardy DJ (2020). Targeting janus kinases and signal transducer and activator of transcription 3 to treat inflammation, fibrosis, and cancer: rationale, progress, and caution. Pharmacol Rev.

[73] Jung YY, Ha IJ, Um JY, Sethi G, Ahn KS (2022). Fangchinoline diminishes STAT3 activation by stimulating oxidative stress and targeting SHP-1 protein in multiple myeloma model. J Adv Res.

[74] Xu G, Yu B, Wang R, Jiang J, Wen F, Shi X (2021). Astragalin flavonoid inhibits proliferation in human lung carcinoma cells mediated via induction of caspase-dependent intrinsic pathway, ROS production, cell migration and invasion inhibition and targeting JAK/STAT signalling pathway. Cell Mol Biol.

[75] Ly JD, Grubb DR, Lawen A (2003). The mitochondrial membrane potential (Deltapsi(m) in apoptosis; an update. Apoptosis.

[76] Brenner D, Mak TW (2009). Mitochondrial cell death effectors. Curr Opin Cell Biol.

[77] Vargason AM, Anselmo AC, Mitragotri S (2021). The evolution of commercial drug delivery technologies. Nat Biomed Eng.

[78] Sreeharsha N, Chitrapriya N, Jang YJ, Kenchappa V (2021). Evaluation of nanoparticle drug-delivery systems used in preclinical studies. Ther Deliv.

[79] Ruan L, Su M, Qin X, Ruan Q, Lang W, Wu M (2022). Progress in the application of sustained-release drug microspheres in tissue engineering. Mater Today Bio.

[80] Wang HC, Pao J, Lin SY, Sheen LY (2012). Molecular mechanisms of garlic-derived allyl sulfides in the inhibition of skin cancer progression. Ann NY Acad Sci.

[81] Wang S, Yu G, Yang W, Wang Z, Jacobson O, Tian R (2021). Photodynamic-Chemodynamic Cascade Reactions for Efficient Drug Delivery and Enhanced Combination Therapy. Adv Sci (Weinh).

