# Supplementary material for: A hybrid nanopharmaceutical for specific-amplifying oxidative stress to initiate a cascade of catalytic therapy for pancreatic cancer
Source: J Nanobiotechnology. 2023 May 24;21:165. doi: 10.1186/s12951-023-01932-0 (PMC10207777; doi:10.1186/s12951-023-01932-0)
Supplement: Supplementary file 1 — Additional file 1: S1. Methods. Figure S1. Size and Zeta potential of SG and SG@M (n = 3). Figure S2. XPS analysis results of SG@M, and the enlarged elements patterns of O, C and Si. Figure S3. UV−vis absorption spectrum of SG and SG@M and photos of the corresponding solutions (inset). Figure S4. Structural formula of galangal (GAL). Figure S5. Standard curve of GAL based on HPLC and chromatogram of GAL content in SG@M. (n = 3). Figure S6. UV-vis absorption spectra of SG@M in PBS (pH=7.2) containing different concentrations of GSH. Figure S7. Mn2+ release from SG@M in various systems. Figure S8. Degraded Si amount from SG in different systems. Figure S9. Degraded Si amount from SG@M in different systems. Figure S10. The trend of absorbance of MB at 665 nm with SG@M and GSH concentration. Figure S11. LSCM images of cellular uptake of SG@M and corresponding flow cytometry analysis. Scale bar: 50 µm. (DiI labeled SG@M NPs and DAPI labeled cell nuclei). Figure S12. Standard curve of ρNA and relative Caspase 9 activity in various treatment groups. (n = 3). Figure S13. Standard curve of ρNA and relative Caspase 3 activity in various treatment groups. (n = 3). Figure S14. Standard Curve of GSH and the concentration trend of SG@M in cellular GSH levels (n = 3). Figure S15. The concentration and time trend in CDT effect of S@M (n=3). Figure S16. Therapeutic efficiency of synergistic effect (SG@M) compared with SG and CDT (S@M). The additive therapeutic efficiencies of independent SG and CDT treatments were estimated using the relation Tadditive = 100 − (fSG × fCDT) × 100, where f is the fraction of surviving cells after each treatment (n=3). Figure S17. The amount of GAL after incubating the cells with free GAL or SG NPs for 4 h and 24 h, respectively (n=3). Figure S18. The uptake levels of free MB and SiO2-MB. (a) LSCM images of PANC-1 cells treated with free MB or SiO2-MB (red fluorescence from MB, blue fluorescence from DAPI-labeled nuclei. Scale bars: 100 µm) and (c) cor [file 12951_2023_1932_MOESM1_ESM.docx]

Supporting Information

A hybrid nanopharmaceutical initiates a cascade of catalytic therapy for pancreatic cancer

Fan Liu^1,2^, Qinyanqiu Xiang^3^, Yuanli Luo^1^, Ying Luo^1^, Wenpei Luo^1^, Qirong Xie^1^, Jingdong Fan^1^, Haitao Ran^1^, Zhigang Wang^1^ and Yang Sun^1^*

1. Department of Ultrasound, The Second Affiliated Hospital of Chongqing Medical University, Chongqing 400010, P. R. China.

2. Chongqing Key Laboratory of Ultrasound Molecular Imaging & State Key Laboratory of Ultrasound in Medicine and Engineering, Chongqing Medical University, Chongqing 400010, P. R. China.

3. Department of Radiology, The First Affiliated Hospital of Chongqing Medical University, Chongqing 400010, P. R. China.

*Corresponding author.

E-mail addresses: sunyang@cqmu.edu.cn

**S1. Methods**

*S1.1* *Loading content of GAL*

10 mg of freeze-dried SG@M was dispersed in 1 mL of dimethyl sulfoxide (DMSO) solution, and the supernatant was collected by centrifugation after left for 24 h to destroy the NP and completely release the drug GAL. The concentration of GAL contained in the supernatant (diluted 100 x) was measured by HPLC. The concentration of GAL was calculated based on the standard curve of GAL, and the loading content (LC) of GAL was calculated according to the following equation [1],

$$LC (\%)=\frac{Weight of loaded GAL}{Weight of SG@M}\times100\%$$

*S1.2 Degraded Si amount*

SG or SG@M (5 mg/mL, 1 mL) was placed into a dialysis bag, which was subsequently placed in 49 mL of PBS (pH 7.2, pH 6.5 with or without GSH (5 mM)). Subsequently, 5 mL of solution outside the dialysis bag was taken at different times (0, 2, 4, 6, 8, 10, 12, 24, and 48 h) and determined by ICP-MS for the concentration of Si.

*S1.3 Intracellular GAL Amount*

PANC-1 cells (1×10^6^ cells per group) were incubated with free GAL (44.36 µg/mL) or SG NPs (400 µg/mL) for 4 h and 24 h, respectively. After several washes of PBS, each group of cells was lysed by three times of freeze-thaw cycle, and the supernatant was collected after centrifugation (16 000 × g) and subjected to HPLC to quantify the concentration of released GAL. The average amount of GAL released per cell was calculated by dividing the total amount of GAL by the number of cells [2].

*S1.4* Preparation of SiO_2_-MB NPs and SiO_2_-DOX NPs

The preparation process is the same as that for SG NPs. Typically, ethanol (75 mL) and ammonia hydroxide (3 mL) were mixed and stirred for 0.5 h. Then, MB (20 mg) or DOX (20 mg) was added into the liquid before stirring for another 0.5 h. Then TEOS (80 µL) was added and stirred for 24 h. SiO_2_-MB and SiO_2_-DOX were obtained by centrifugation at 13 000 rpm for 10 min.

*S1.5 Cell uptake of free MB,* *SiO_2_-MB NPs, free DOX and SiO_2_-DOX NPs*

PANC−1 cells were exposed to free MB, SiO_2_-MB NPs, free DOX and SiO_2_-DOX NPs for 4 h and 24 h. All cells were washed with PBS before the fluorescent images were obtained by LSCM (MB: λ_ex_ = 633 nm, λ_em_ = 650-750 nm; DOX: λ_ex_ = 552 nm, λ_em_ = 555-650 nm). Flow cytometry measured the intracellular mean fluorescence intensity (MFI).

**S2. Results**

**
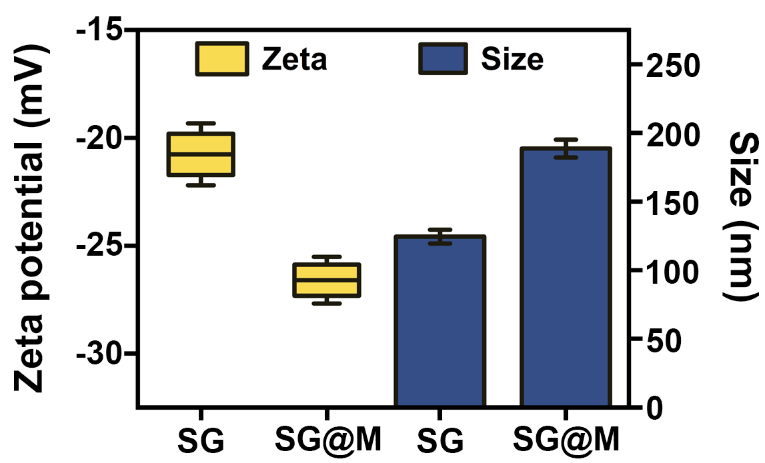
**

**Figure S1.** Size and Zeta potential of SG and SG@M (n = 3).


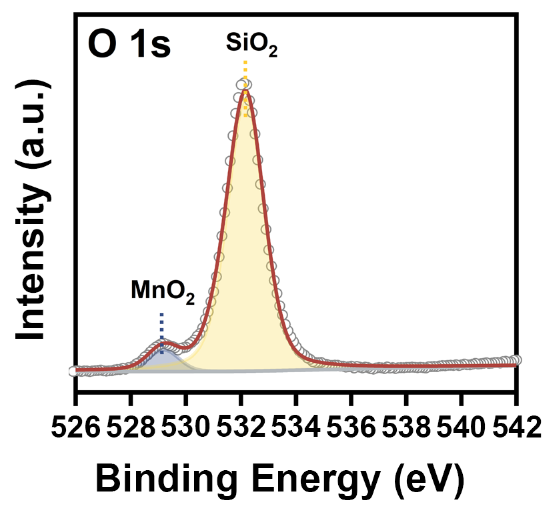

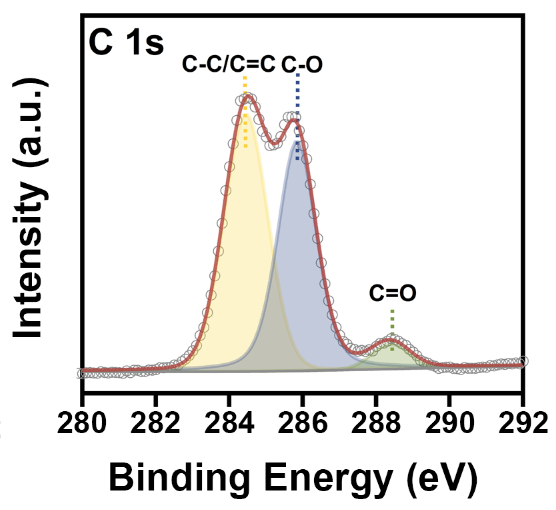

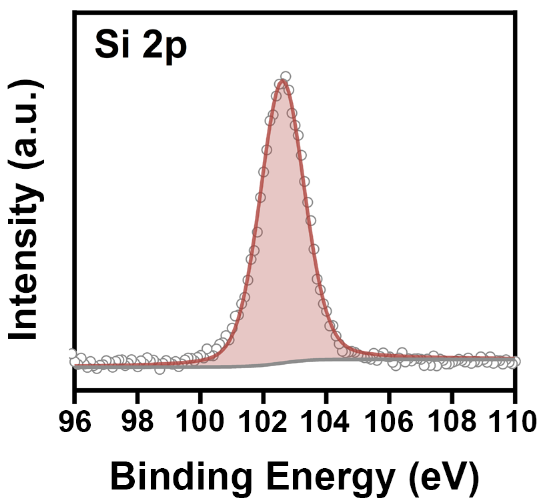


**Figure S2.** XPS analysis results of SG@M, and the enlarged elements patterns of O, C and Si.

**
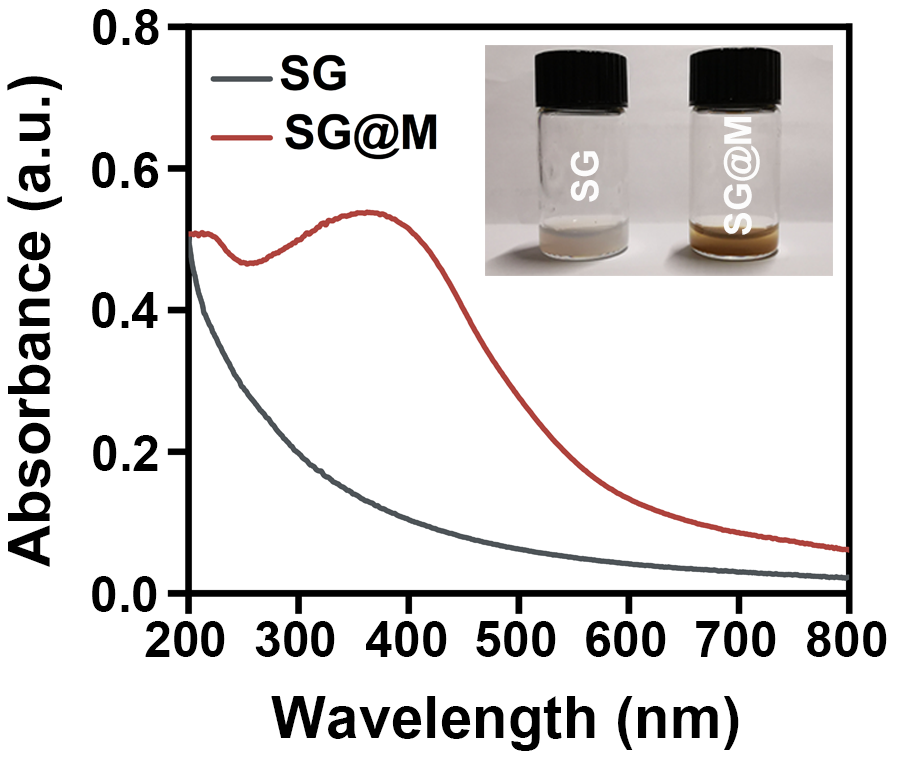
Figure S3.** UV−vis absorption spectrum of SG and SG@M and photos of the corresponding solutions (inset)

**
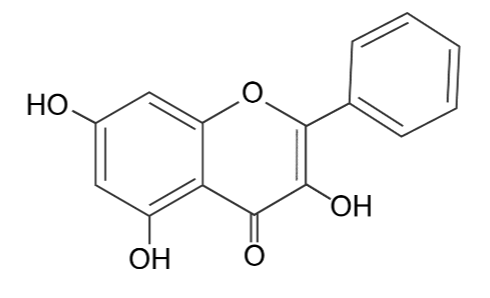
**

**Figure S4.** Structural formula of galangal (GAL).

**
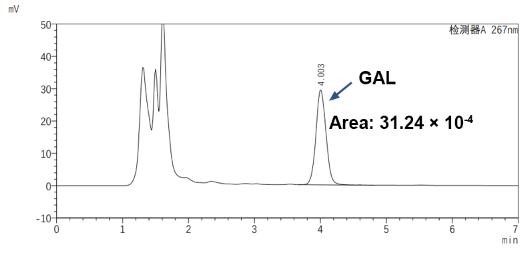

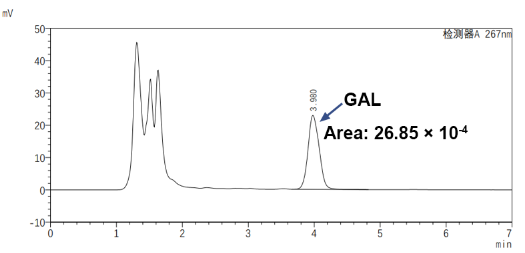

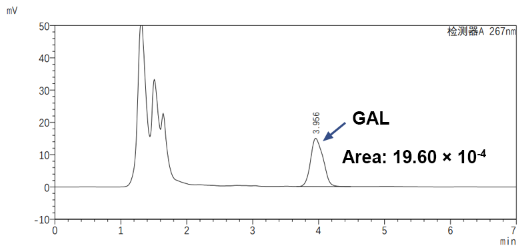

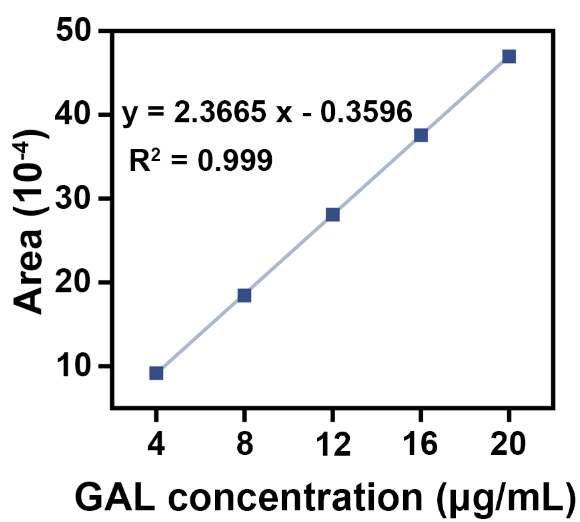
Figure S5.** Standard curve of GAL based on HPLC and chromatogram of GAL content in SG@M. (n = 3)

**Figure S6.** UV-vis absorption spectra of SG@M in PBS (pH=7.2) containing different concentrations of GSH.

**
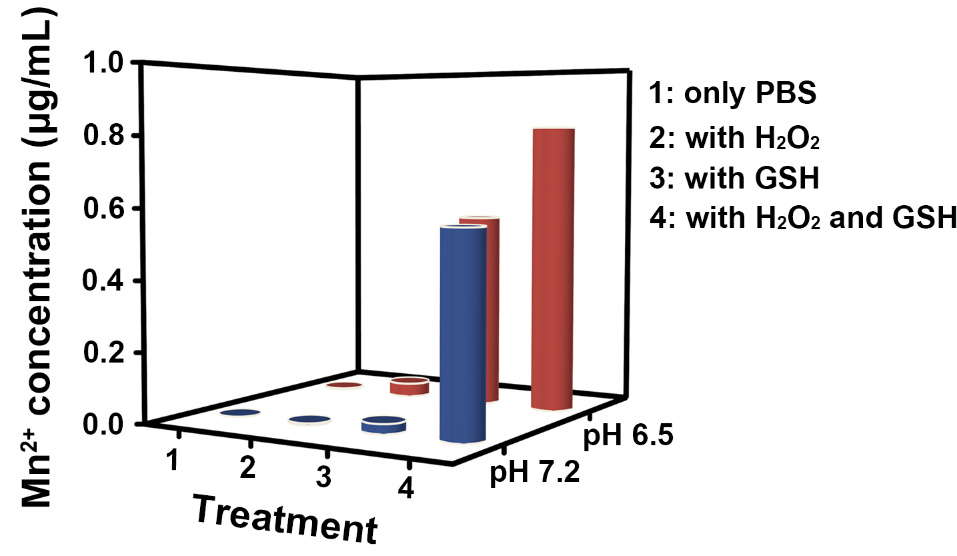
****Figure S7.** Mn^2+^ release from SG@M in various systems.


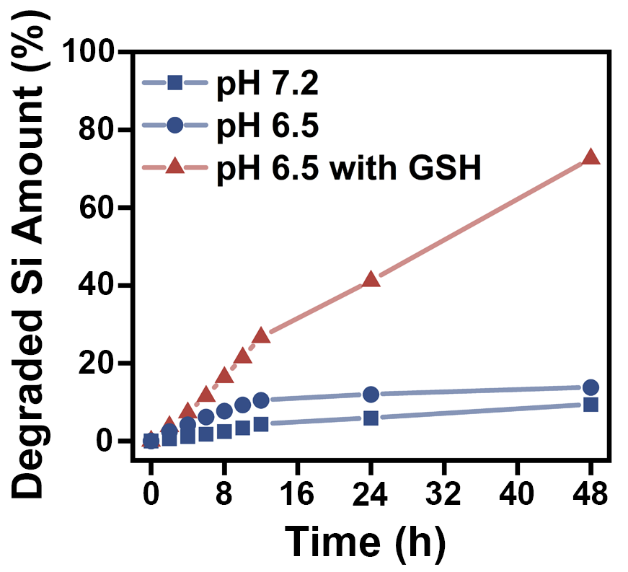
**Figure S8.** Degraded Si amount from SG in different systems.

**Figure S9.** Degraded Si amount from SG@M in different systems.

**
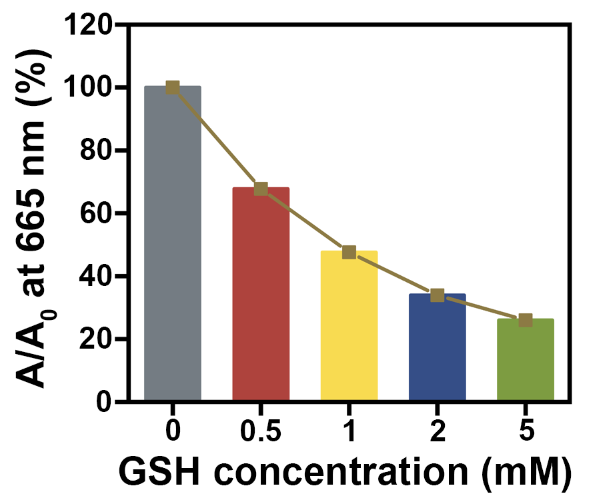

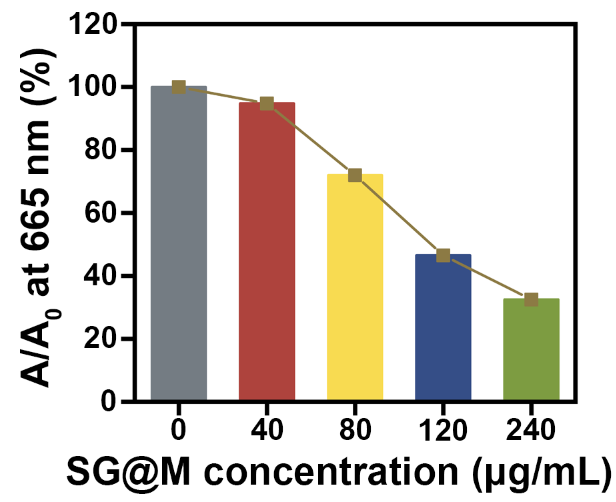
**

**
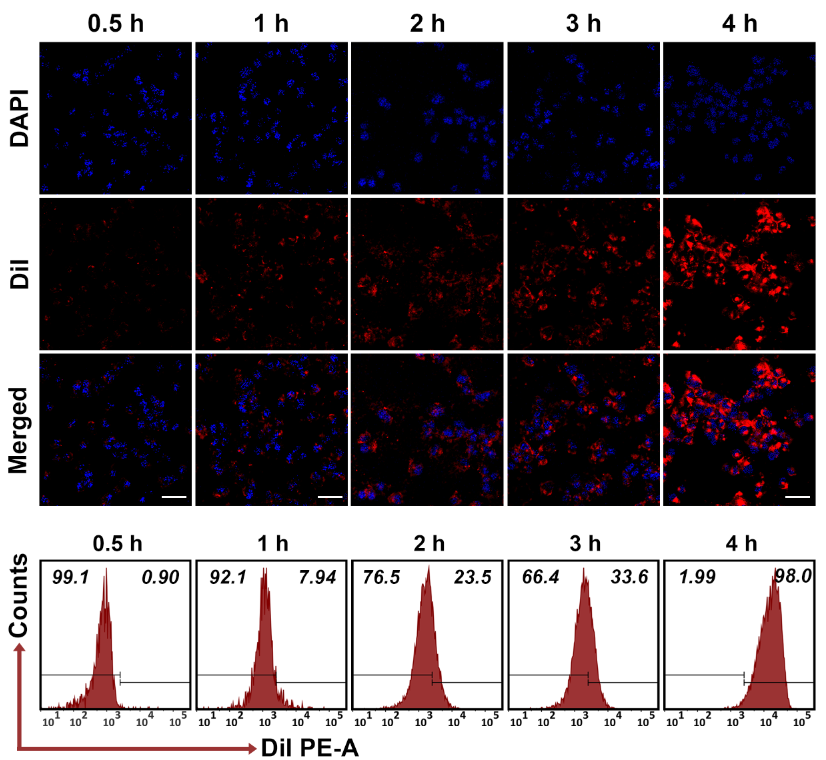
Figure S10.** The trend of absorbance of MB at 665 nm with SG@M and GSH concentration.

**Figure S11.** LSCM images of cellular uptake of SG@M and corresponding flow cytometry analysis. Scale bar: 50 µm. (DiI labeled SG@M NPs and DAPI labeled cell nuclei)

**
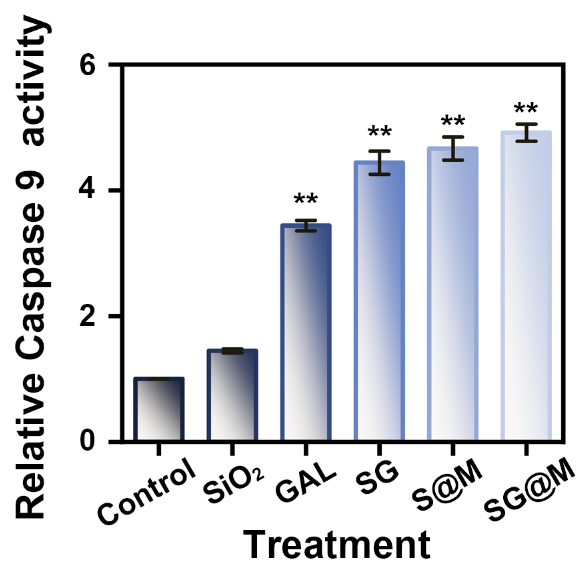

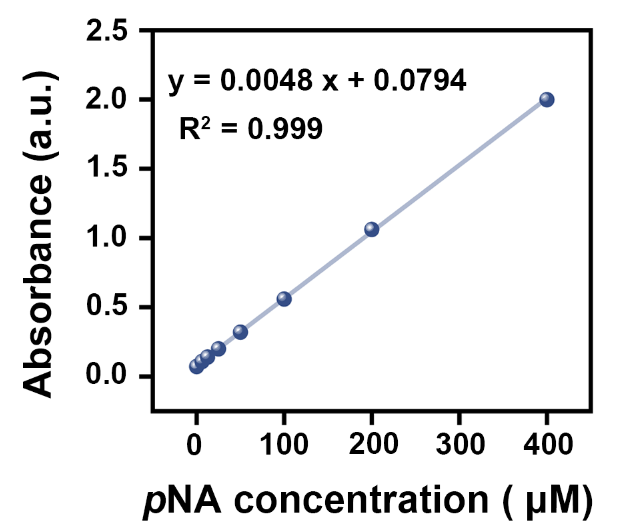
**

**Figure S12.** Standard curve of *ρ*NA and relative Caspase 9 activity in various treatment groups. (n = 3).


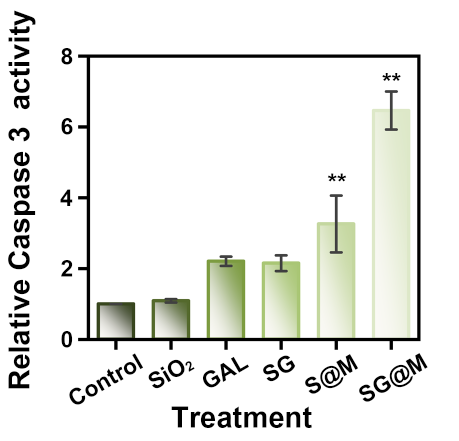
**
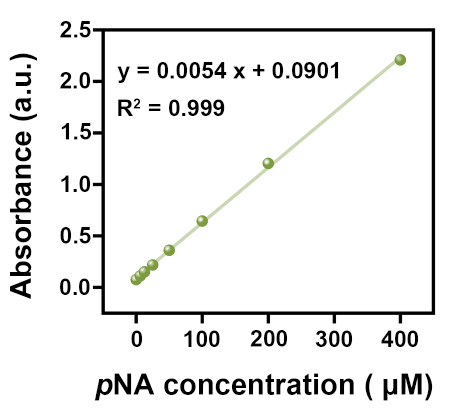
**

**Figure S13.** Standard curve of *ρ*NA and relative Caspase 3 activity in various treatment groups. (n = 3)*.*

**
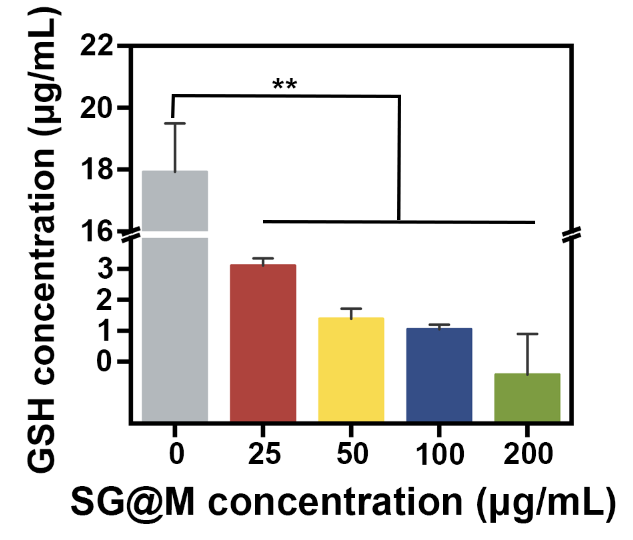

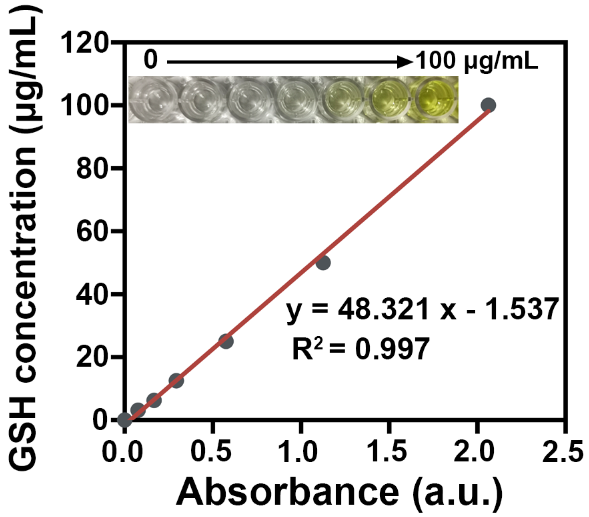
**

**
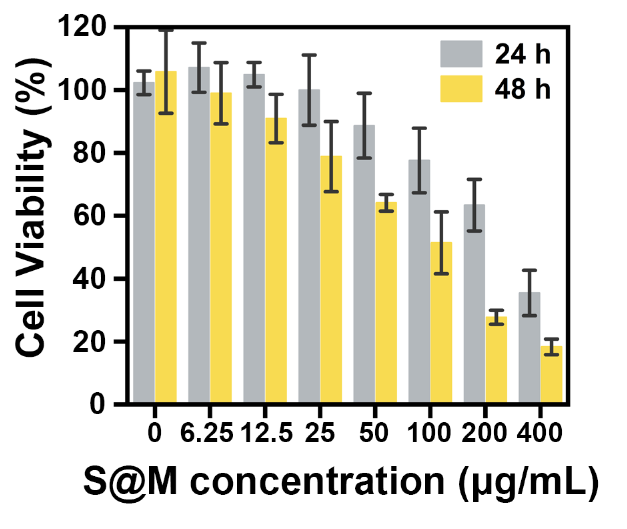
Figure S14.** Standard Curve of GSH and the concentration trend of SG@M in cellular GSH levels (n = 3).

**Figure S15.** The concentration and time trend in CDT effect of S@M (n=3).

**Figure S16** Therapeutic efficiency of synergistic effect (SG@M) compared with SG and CDT (S@M). The additive therapeutic efficiencies of independent SG and CDT treatments were estimated using the relation *T_additive_* = 100 − (*f_SG_* × *f_CDT_*) × 100, where *f* is the fraction of surviving cells after each treatment (n=3).

**Figure S17** The amount of GAL after incubating the cells with free GAL or SG NPs for 4 h and 24 h, respectively (n=3).

**
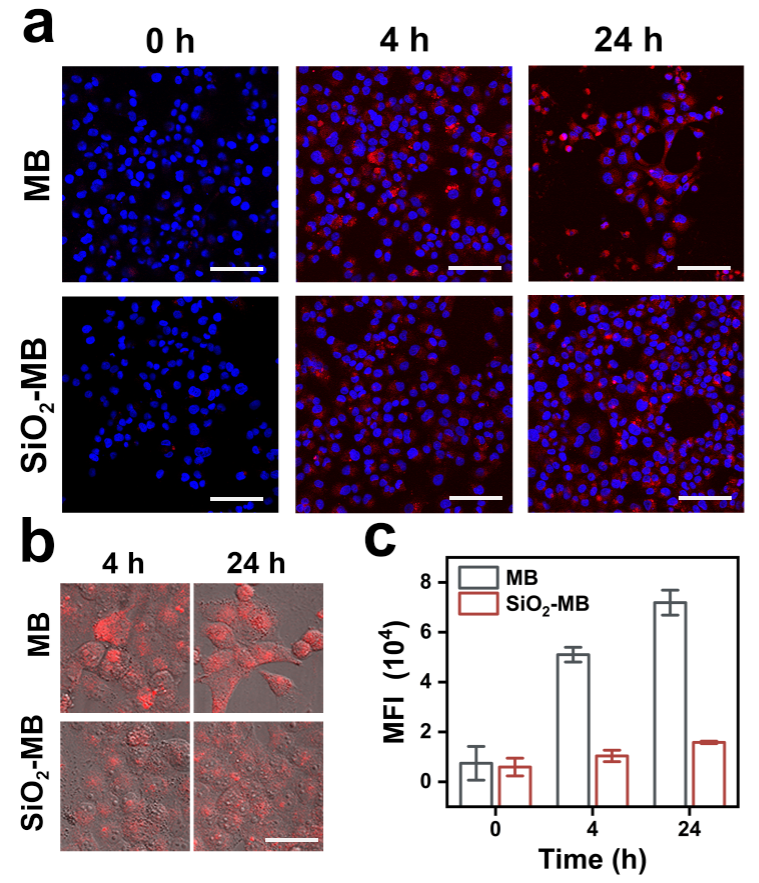
Figure S18** The uptake levels of free MB and SiO_2_-MB. (a) LSCM images of PANC-1 cells treated with free MB or SiO_2_-MB (red fluorescence from MB, blue fluorescence from DAPI-labeled nuclei. Scale bars: 100 µm) and (c) corresponding transmittance images the morphologies of the specific cells (Scale bar: 50 µm). (c) Corresponding flow cytometry analysis (n=3).

**
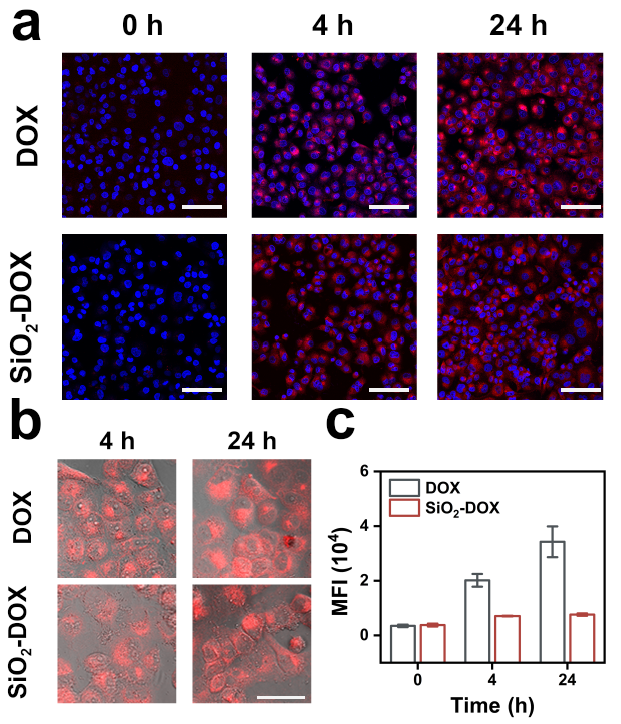
**

**Figure S19** The uptake levels of free DOX and SiO_2_-DOX. (a) LSCM images of PANC-1 cells treated with free DOX or SiO_2_-DOX (red fluorescence from DOX, blue fluorescence from DAPI-labeled nuclei. Scale bars: 100 µm) and (c) corresponding transmittance images the morphologies of the specific cells (Scale bar: 50 µm). (c) Corresponding flow cytometry analysis (n=3).

**
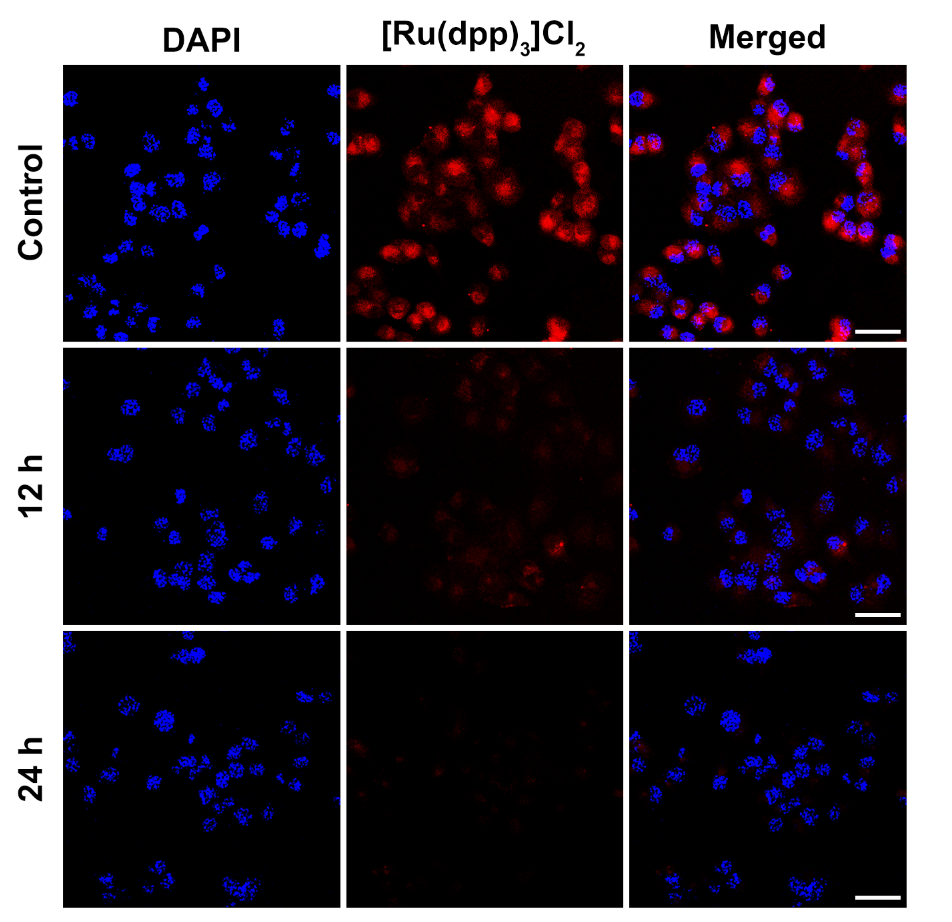
Figure S20.** LSCM images of O_2_ production detected with RDPP after 12 h and 24 h incubation with SG@M. Scale bars: 50 µm.

**References**

1. Liu J, Zhang J, Song K, Du J, Wang X, Liu J, et al. Tumor Microenvironment Modulation Platform Based on Composite Biodegradable Bismuth-Manganese Radiosensitizer for Inhibiting Radioresistant Hypoxic Tumors. Small. 2021;17(34):e2101015.

2. Zhang S, Chu Z, Yin C, Zhang C, Lin G, Li Q. Controllable drug release and simultaneously carrier decomposition of SiO2-drug composite nanoparticles. J Am Chem Soc. 2013;135(15):5709-16.
